# Phylogeny, Age, and Evolution of Tribe Lilieae (Liliaceae) Based on Whole Plastid Genomes

**DOI:** 10.3389/fpls.2021.699226

**Published:** 2022-02-01

**Authors:** Juan Li, Jing Cai, Huan-Huan Qin, Megan Price, Zhen Zhang, Yan Yu, Deng-Feng Xie, Xing-Jin He, Song-Dong Zhou, Xin-Fen Gao

**Affiliations:** ^1^Key Laboratory of Bio-Resources and Eco-Environment of Ministry of Education, College of Life Sciences, Sichuan University, Chengdu, China; ^2^Sichuan Key Laboratory of Conservation Biology on Endangered Wildlife, College of Life Sciences, Sichuan University, Chengdu, China; ^3^CAS Key Laboratory of Mountain Ecological Restoration and Bioresource Utilization & Ecological Restoration and Biodiversity Conservation Key Laboratory of Sichuan Province, Chengdu Institute of Biology, Chinese Academy of Sciences, Chengdu, China

**Keywords:** *Lilium*, *Fritillaria*, divergence time estimate, QTP, Liliales, trait evolution

## Abstract

Tribe Lilieae, encompassing *Lilium*, *Notholirion*, *Cardiocrinum*, and *Fritillaria*, includes economically important crops with a horticultural and medicinal value. It is considered to be a core lineage of Liliaceae, but phylogenetic relationships within it, and the timing of the origin of individual clades, remain incompletely resolved. To address these issues, we reconstructed the evolutionary history of the tribe. We sequenced 45 Liliaceae plastomes and combined them with publicly available data (for a total of 139 plastomes) to explore the systematics, origin, divergence, and evolution of Lilieae. Our taxon sampling covers all ten sections of *Lilium*, all *Cardiocrinum* species, three *Notholirion* species, and major phylogenetic clades of *Fritillaria*. Our phylogenetic analysis confirms the monophyly of major sections/subgenera of *Lilium* and *Fritillaria* with strong support. We dated the origin of Lilieae to the Eocene, with genera and species radiations inferred to have occurred in the Miocene. The reconstruction of the ancestral area implies that Lilieae may have originated from the Qinghai-Tibet Plateau (QTP): the Himalayas and Hengduan Mountains and uplifting of the QTP likely promoted divergence within the tribe. Ancestral-state reconstructions of the bulb component number (including bulblets and scales) show a strong correlation with the genus-level phylogenetic diversity in Lilieae. They also predict that the most recent common ancestor of Lilieae had bulbs with numerous bulblets. Based on these observations, we predicted that climatic oscillations associated with the QTP uplift played an important role in the evolution of the Lilieae bulb. Our findings provide a well-supported picture of evolutionary relationships and a useful framework for understanding the pathway of bulb evolution within Lilieae, contributing to a better understanding of the evolutionary history of lilies.

## Introduction

Liliaceae (Liliales) have a substantial importance in the systematic history of the monocotyledons, and the family itself has a complicated taxonomic history (Cronquist, 1988; Chase et al., 1995; APG, 1998, 2003, 2009, 2016; Patterson and Givnish, 2002; Givnish et al., 2016, 2018, 2020; Peruzzi, 2016; Kim and Kim, 2018). The classification of the family has changed substantially in the last three decades (Patterson and Givnish, 2002; Peruzzi et al., 2009; Givnish et al., 2016; Peruzzi, 2016; Kim and Kim, 2018). Tribe Lilieae is considered to be a core member of Liliaceae and encompasses four closely related genera, namely, *Lilium* (including *Nomocharis*, which was accommodated in *Lilium*; Gao and Gao, 2016), *Notholirion*, *Cardiocrinum*, and *Fritillaria* (Gao et al., 2013; APG, 2016; Peruzzi, 2016). Lilieae has 260 species distributed in northern hemisphere temperate regions (Boufford, 1993; Chen et al., 2000), including many well-known horticultural and medicinal species. For example, longiflorum lily (*L. longiflorum*), orange lily (*L. bulbiferum*), and Crown Imperial (*F. imperialis*) are widely cultivated (Pelkonen et al., 2010; Wietsma et al., 2015; Tuyl et al., 2018), and the bulbs of *F. cirrhosa* and *F. thunbergii*, known as “beimu,” are used in traditional Chinese medicine (Chen et al., 2000; Wang et al., 2009). Despite the importance of lilies, the origin, divergence, and evolution of Lilieae are still quite poorly understood.

The species of Lilieae are herbaceous geophytes with rounded bulbs. They have showy flowers with six free tepals and stamens, dorsifixed anthers, and a trilocular superior ovary (Chen et al., 2000; Patterson and Givnish, 2002). Within Lilieae, *Cardiocrinum* species have cordate leaves (Ohara et al., 2006), and *Notholirion* species have numerous bulblets and a raceme inflorescence (Li et al., 2020). *Fritillaria* generally differs from *Lilium* in that their bulbs comprise two or three farinaceous scales and nodding solitary flowers (Mustafa and Abdul-Razaq, 2015). Within *Lilium*, the bulbs have many fleshy scales, and the flowers are quite diverse in shape and ornamentation (Comber, 1949; Hayashi and Kawano, 2000). Thus, members of Lilieae vary in leaves, flowers, and bulbs, all features of considerable taxonomic and evolutionary significance. However, the evolution of these morphological characteristics is still poorly understood (Peruzzi, 2016; Huang et al., 2018).

Previous studies have confirmed the monophyly of Lilieae (Patterson and Givnish, 2002; Fay et al., 2006; Kim et al., 2013; Petersen et al., 2013; Givnish et al., 2016; Kim and Kim, 2018; Do et al., 2020). However, these studies only involved a small number of Lilieae species. Phylogenetic relationships between the two large genera in Lilieae, namely, *Fritillaria* and *Lilium*, have been controversial with observed phylogenetic incongruences and a lack of strong support when taxon sample sizes were increased (Hayashi and Kawano, 2000; Ronsted et al., 2005; Lkinci et al., 2006; Huang et al., 2018). In previous phylogenetic examination by Huang et al. (2018) that involved more than half of all Lilieae species, *Fritillaria* was divided into two clades, and *Lilium* was nested within *Fritillaria*. Similarly, the infrageneric classifications of *Fritillaria* and *Lilium* have been much studied (Hayashi and Kawano, 2000; Wang et al., 2009; Muratovic et al., 2010; Lee et al., 2011; Gao et al., 2013; Day et al., 2014; Mustafa and Abdul-Razaq, 2015; Givnish et al., 2020). *Fritillaria* is currently divided into eight subgenera that are generally supported by phylogenetic analyses (Rix, 2001; Ronsted et al., 2005; Day et al., 2014). For *Lilium*, phylogenetic studies are often built on the seven morphological sections of Comber (1949). Nonetheless, previous studies focused on partial clades and lack strong support, and so details of the infrageneric relationships for *Fritillaria* and *Lilium* remain controversial (Gao et al., 2013; Du et al., 2014; Ghanbari et al., 2018; Tuyl et al., 2018; Givnish et al., 2020). Thus, with an increasing phylogenetic conflict being published, the resulting uncertainty in overall Lilieae phylogeny (Huang et al., 2018; Kim and Kim, 2018) has limited further evolutionary study of the tribe.

A key difficulty in Lilieae phylogenetic reconstruction relates to the development or application of suitable molecular markers for addressing these issues. Until recently, the nuclear ribosomal internal transcribed spacer (ITS) sequence was widely used to reconstruct phylogenetic relationships in Liliaceae (Lkinci et al., 2006; Muratovic et al., 2010; Lee et al., 2011; Nursel, 2011; Gao et al., 2013; Du et al., 2014; Ghanbari et al., 2018). However, most ITS-based clades are weakly supported and so may not adequately reconstruct the phylogeny and evolution of Lilieae. Similarly, individual plastid regions or genes (i.e., one or a few) have been widely applied in molecular phylogenetic studies of Liliales families and genera (Patterson and Givnish, 2002; Ronsted et al., 2005; Petersen et al., 2013; Mustafa and Abdul-Razaq, 2015; Kiani et al., 2017; Givnish et al., 2020), and they also did not provide fully clear relationships in Lilieae, especially in *Fritillaria* and *Lilium* (Ronsted et al., 2005; Day et al., 2014; Huang et al., 2018).

Using the complete plastid genome (i.e., plastome) much more detailed molecular information data than traditional nuclear or plastid markers (e.g., Goremykin et al., 2003; Leebens-Mack et al., 2005; Raman and Park, 2016; Xie et al., 2018; Guo et al., 2020) are provided. The data from plastomes have been widely used to infer the phylogenetic relationships and phylogeographic histories of plant taxa considering their relatively small size, conservation of gene content and order, and effectively high copy number (Givnish et al., 2010, 2016, 2018; Raman and Park, 2016; Sancho et al., 2017; Li et al., 2019; Xie et al., 2019, 2020b; Guo et al., 2020). Given technological advances in sequencing, recent studies have used plastomes to resolve phylogenetic uncertainties in monocotyledons (Givnish et al., 2010, 2016, 2018, 2020; Barrett et al., 2013, 2014; Mennes et al., 2015; Ross et al., 2016; Lam et al., 2018; Liu et al., 2018; Xie et al., 2018; Soto Gomez et al., 2020; Su et al., 2021). Here, we sequenced 45 Liliaceae plastomes and combined 94 publicly available plastomes to explore the origin, divergence, and evolution of Lilieae. Our study determines (1) whether *Lilium* and *Fritillaria* are monophyletic, (2) when and how Lilieae originated and diverged, and (3) the history of morphological trait evolution in Lilieae.

## Materials and Methods

### Taxon Sampling

We included 139 plastid genomes of monocots representing nine families of Liliales and 11 monocot outgroups (GenBank accessions: Supplementary Table 1). We studied 79 species of Lilieae, which encompassed three of four *Notholirion* species (excluding *N. koeiei*), all three *Cardiocrinum* species, 26 of 152 *Fritillaria* species, and 47 of 118 *Lilium* species (Supplementary Table 1). Samples covered all ten sections of *Lilium* and major phylogenetic clades (six of eight subgenera) of *Fritillaria* (Comber, 1949; Ronsted et al., 2005; Gao et al., 2013; Day et al., 2014). Among all 139 plastomes, we assembled 45 plastomes, with this study including the first publication of molecular data from *F. fusca*. Fresh material from adult plants was collected in the field between 2009 and 2020 and dried in the silica gel for further DNA extraction. Voucher specimens are deposited in the Sichuan University Herbarium [SZ, the Herbarium code refers to Thiers (2016)]. Detailed information of materials included in this study is listed in Supplementary Table 1. In addition, we downloaded 82 ITS sequences of Lilieae species from GenBank.

### DNA Sequencing and Genome Assembly

We extracted total genomic DNAs from 100 mg desiccated leaves using a modified cetyltrimethylammonium bromide method (Doyle, 1987). Total genomic DNAs were sent to Novogene Technologies, Inc. (Beijing, China) for genome library construction and sequencing. The sequencing library was generated using NEB Next^®^ Ultra™ DNA Library Prep Kit for Illumina (NEB, United States) following the recommendations of the manufacturer, and index codes were added to each sample. Sequencing was performed using an Illumina Novaseq2500 sequencer (Illumina, San Diego, CA, United States).

The plastomes were *de novo* assembled using the NOVOPlasty 2.7.2 program (Dierckxsens et al., 2017) with raw data. To minimize the impact of distant starting seed sequences on the plastomes, we used a consistent seed sequence within each genus, specifically *L. henryi* (NC035570), *F. cirrhosa* (MH244906), *N. macrophyllum* (MH011354), *Tulipa sylvestris* (MT261172, which was also used as a seed of *Lloydia tibetica*), and *Smilax china* (HM536959). Genome annotation and IR region searches were processed using PGA software (Qu et al., 2019). Manual modifications for the uncertain start and stop codons were conducted based on comparison with homologous genes from other plastomes of other species using GENEIOUS R11 (Kearse et al., 2012).

### Phylogenetic Analyses

We reconstructed phylogenetic relationships based on the data of Lilieae species (including a 79-taxon plastome data set and a separate data set comprising 77 nuclear ITS sequences) to analyze the phylogeny of Lilieae, using five species from the tribe Tulipeae as an outgroup in each case. For plastomes, all sequences including protein-coding genes (CDS), ribosomal RNA genes (rDNA), transfer RNA genes (tRNA), and non-coding regions (NCR) were extracted using the PhyloSuite platform (Zhang et al., 2020) and then aligned using the multiple alignments using Fast Fourier transform (MAFFT) program (Katoh et al., 2005). Because *infA*, *ycf1*, *ycf15*, and *ycf68* have a high-sequence variability, we excluded these genes from the analysis (Givnish et al., 2018; Li et al., 2019; Lu et al., 2021). We adjusted all alignments manually using the GENEIOUS R11 software (Kearse et al., 2012) and then concatenated them into supermatrices using the PhyloSuite platform (Zhang et al., 2020). We created two matrices, namely, CDS matrix (i.e., CDSs only) and a whole plastid supermatrix (WP: CDS + tRNA + rDNA + NCR).

We used the PartitionFinder 2 program (Lanfear et al., 2017) to determine optimal partitions for plastomes by the Akaike information criterion method, considering each gene or individual non-coding sequence as an initial partition. A total of 57 partitions of the WP were designated for the across-Lilieae analysis; 41 had a generalized time-reversible (GTR) + I model, 12 had a GTR + G model, and the remaining four had a GTR model (Supplementary Table 2). We performed partitioned maximum likelihood (ML) analyses using the IQ-TREE v1.6.11 program (Nguyen et al., 2015). The ML analyses of ITS were performed using the RAxML v.8 software (Stamatakis, 2014) under the GTR + G model selected by jModelTest (Posada, 2008). All the ML analyses used 1000 rapid-search bootstrap replicates. Bayesian inference (BI) was performed using the MrBayes v3.2 software (Ronquist et al., 2012). The models of partitions and jModelTest (the same set used in the likelihood analyses) for BI analyses were parameterized independently. Two independent runs of 10 million generations were performed using the BI analyses. Trees were sampled once every 1,000 generations with the first 25% trees of each run discarded as the burn-in. The stationarity was reached when the average standard deviation (SD) of split frequencies remained below 0.001 and effective sample size (ESS) >200.

### Estimation of Divergence Time

There are currently no well-documented fossils in Liliaceae, and thus fossil constraints were limited to Liliales. We used 128 Liliales plastomes (more taxa than for the main phylogenetic analyses, which focused on Lilieae) and 11 additional outgroup plastomes (Supplementary Table 1) to estimate the origin times of Lilieae and other allied taxa. Referring to previous studies (Li et al., 2019; Xie et al., 2020a) and to minimize the effects of missing data, we only used combined single-copy CDS genes data set derived from 139 plastomes for the estimation of divergence time. The data set included nine of ten families of Liliales (excluding mycoheterotrophic Corsiaceae) and all tribes (including 12 genera) of Liliaceae. Estimations of divergence time were performed using an uncorrelated lognormal relaxed molecular clock method implemented in the BEAST 1.10.4 program (Suchard et al., 2018); a Yule process (Gernhard, 2008) was specified as the tree prior. An optimal partitioning scheme was determined using the PartitionFinder 2 program (Lanfear et al., 2017). The information for model settings is presented in Supplementary Table 3. Six calibration points (i.e., four from fossils) were used to calibrate time as follows (Supplementary Table 4; see Iles et al., 2015; Givnish et al., 2016; Huang et al., 2018). (1) Based on the fossilized leaves of *Luzuriaga peterbannisteri* (Conran et al., 2014), the crown node of *Luzuriaga* (*Drymophila*/*Luzuriaga* clade: Alstroemeriaceae) was constrained to a minimum age of 23.2 Ma (Lindqvist and Lee, 2009; Conran et al., 2014). (2) The stem node of Ripogonaceae was constrained to a minimum age of 51.5 Ma (Carpenter et al., 2007; Conran et al., 2009) based on the fossilized leaves of *Ripogonum tasmanicum* (Conran et al., 2009). (3) Based on previous studies about fossilized seeds of *Spirematospermum chandlerae* (Friis, 1988; Iles et al., 2015; Givnish et al., 2018), the crown node of Zingiberales was set as 83 Ma. (4) Based on the fossilized leaves of *S. trinervis* (Huzioka, 1963), which is close to *S. china* (Morita, 1932), the stem node of *S. china* clade was set as 7.2–5.33 Ma age (Denk et al., 2015). These four fossil prior calibrations were set as the mean in lognormal distributions with an SD of 0.5 million years. More details about parameters related to calibration points are shown in Supplementary Table 4. (5) Referring to the review of Chacón et al. (2012), the crown node of *Smilax* was set to 46 Ma [95% highest probability density (HPD) 54.8–37.2 Ma, a lognormal prior distribution], which represents a conservative minimal age. This considered that *Smilax*-like fossils are known from the Early/Lower Eocene (55.8–48.6 Ma; Edelman, 1975; Wilf, 2000) and the Middle Eocene (48.6–37.2 Ma; MacGinitie, 1941; Wilde and Frankenhauser, 1998). (6) According to previous research of the evolutionary timescale of monocots (Givnish et al., 2016), 123.8 Ma was implemented as a minimum age of Liliales and as the zero offset of lognormal distribution with a log mean of 123 (95% HPD: 131.1–115.6) and SD of 4.3 in the uncorrelated lognormal analysis. This time is equal to the stem group of the Liliales and is congruent with several studies about Liliales/monocots (Eguchi and Tamura, 2016; Barba-Montoya et al., 2018; Givnish et al., 2018; Xie et al., 2020a). We ran an empty analysis without the data to evaluate the interactions of the priors first. The result of the empty run produced reasonable ages of the calibrated nodes according to the fossils. For each BEAST analysis, the Markov chain Monte Carlo (MCMC) algorithm was run for 100 million generations with sampling every 10,000 generations, followed with a burn-in of the initial 10% cycles. MCMC samples were inspected in TRACER to confirm sampling adequacy and convergence of the chains to a stationary distribution.

### Reconstruction of Ancestral Area

The following five regions were defined for biogeographic analyses based on the paleogeographic and climatic evidence (Lkinci et al., 2006; Tu et al., 2010; Buerki et al., 2012) and also according to the distribution of Lilieae: (A) the Qinghai-Tibet Plateau-Himalayas-Hengduan Mountains (QTP-HHM), (B) East Asia and Siberia, (C) Northern America, (D) Irano-Turanian region (central and western Asia, north-east Africa, and north-west China), and (E) Europe and the Mediterranean Basin. All these regions are separated by physical barriers or climatological differences. Reconstructions of the ancestral area of Lilieae were conducted using the Statistical Dispersal-Vicariance (S-DIVA) analysis as implemented in the RASP v4 software (Yu et al., 2020). We used the BI tree based on the WP data (obtained by phylogenetic analyses) for the S-DIVA analyses. We tested the implemented biogeographical models DEC, DIVALIKE, and BAYAREALIKE with and without the J-parameter modeling jump dispersal (Matzke, 2013). The BAYAREALIKE + J model for biogeographical reconstruction yielded the best model fit. Because uncertainty in the root areas of an outgroup can lead to biased inferences for the crown node of the ingroups (Yu et al., 2015), we removed outgroups before ancestral-state reconstruction. To explore the effects of area constraints, the maximum number of areas at each node was set to three.

### Ancestral Character-State Reconstructions

Lilieae species vary in traits associated with bulbs, flowers, and leaves that have considerable ecological and evolutionary significance (Stevenson and Loconte, 1995; Patterson and Givnish, 2002; Peruzzi et al., 2012). We conducted reconstructions of seven vegetative features, namely, (i) bulb component (including bulblets and scales) number, (ii) stem height, (iii) leaf length, (iv) leaf width, (v) flower number per inflorescence, (vi) tepal length, and (vii) tepal width, all based on field observations, specimen study, and information in the literature (Shoe, 1958; Boufford, 1993; Chen et al., 2000; Ohara et al., 2006; Li et al., 2020). The details of the seven characters are provided in Supplementary Table 5. All trait measurements of specimens were carried out using MATO (Altınordu et al., 2016). We used the mean values of seven traits as qualitative character states. The Comparison Trees and States method in the RASP v4 software (Yu et al., 2020) was used to assess the phylogenetic signal in the seven traits. The phylogenetic signal is the tendency of related species to resemble each other in a specific character more than species drawn at random from the same tree (Munkemuller et al., 2012; Yu et al., 2020). After phylogenetic signal analyses, we found that the bulb component number was the only character to show a strong phylogenetic signal (see below). We conducted the reconstruction of an ancestral trait of the bulb type using MultiState Reconstruction with the Bayes Traits method implemented in RASP. Lilieae bulbs were divided into three types, namely, (i) few scales (scale numbers < 5), (ii) many scales (scale numbers ≥ 5), and (iii) numerous bulblets, coded as A, B, and C, respectively, in Supplementary Table 5 (letter line). The MCMC iterations were set as 100 million, sampled every 10,000, and the first 50,000 iterations were set as burn-in.

## Results

### Plastome Features and Sequence Divergence of Lilieae

Plastome features are conserved (Supplementary Table 6) among the 80 included plastomes (79 species) of Lilieae. Their total length ranges from 151,009 bp (*F. ussuriensis*) to 153,235 bp (*L. fargesii*); their GC content is very similar (36.9–37.1%). There are 115 unique genes, comprising 81 CDS genes, 4 rDNA genes, and 30 tRNA genes (of these, nine, four, and eight genes, are located in the inverted repeats). We obtained 53 non-coding regions excluding lengths of less than 200 bp. The length of single sequences ranged from 71 bp (*trnG-UCC*) to 6,660 bp (*ycf2*).

### Phylogenetic Analysis

**FIGURE 1:**
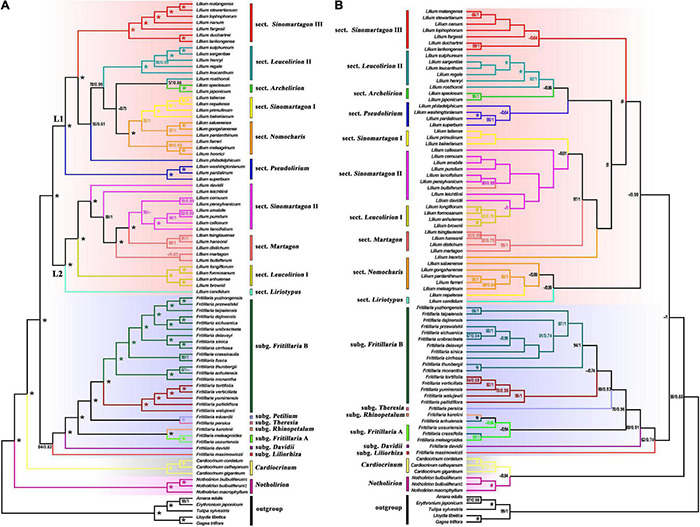
Phylogenetic relationships of Lilieae species inferred using whole plastomes (WP) and nuclear ribosomal internal transcribed spacer (ITS) sequence data. Maximum-likelihood bootstrap support/Bayesian posterior probability values are shown near corresponding nodes (“-” indicates support values less than 50%; “*” represents 100%/1 support). The genus, subgenus, and sectional classifications of Lilieae are indicated to the middle of trees and colored within trees. **(A)** Phylogenetic relationships inferred from WP data and **(B)** Phylogenetic relationships inferred from ITS data.

For plastomes, the topologies from ML (Supplementary Figures 1, 2) and BI (Figure 1A and Supplementary Figure 3) trees are congruent, and from here on, we referred to the BI tree (Figure 1A). The trees reconstructed from CDSs (Supplementary Figures 2, 3) and WP matrices (Figure 1A and Supplementary Figure 1) were topologically consistent with each other with little difference in well-supported branches in terms of posterior probabilities (PP) or bootstrap support values (BS). The ITS tree (Figure 1B and Supplementary Figure 4) is roughly comparable to the WP tree regarding intergeneric relationships but is weakly supported regarding subgeneric clades (cf. Figures 1A,B). In all analyses, Lilieae is monophyletic. In the WP tree (Figure 1A), four genera are monophyletic with robust support (*PP* = 1.00, *BS* = 100), and *Fritillaria* is monophyletic with moderate support (*PP* = 0.82, *BS* = 84). *Fritillaria* comprises seven subgenera, which all have robust support (Figure 1A). *Lilium* is divided into two main lineages (marked as “L1” and “L2” in Figure 1A) with strong support. *Lilium* species are divided into ten sections based on morphology and molecular results following previous studies on *Lilium* (Comber, 1949; Nursel, 2011; Gao et al., 2013; Du et al., 2014; Huang et al., 2018; Tuyl et al., 2018), but only four sections (i.e., *Leucolirion* I, *Sinomartagon* I and III, *Archelirion*) are inferred to be monophyletic here.

### Estimation of Divergence Time and Reconstruction of Ancestral Area

**FIGURE 2:**
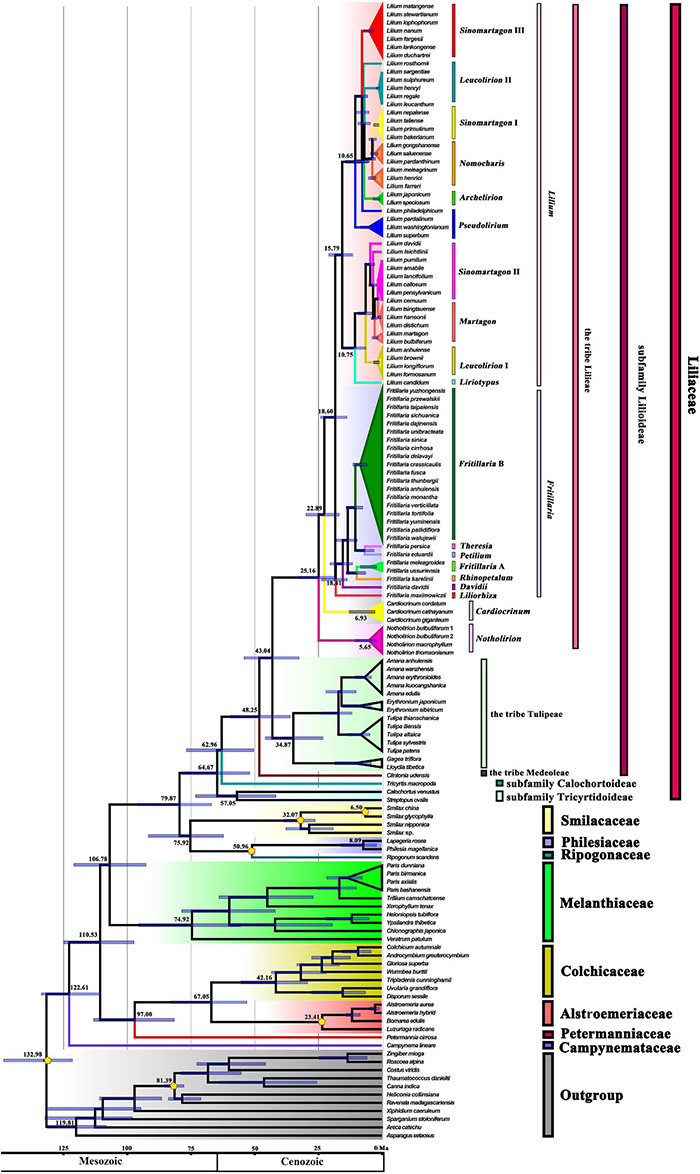
The estimation of divergence time based on 139 plastomes of Liliales and outgroups. The 95% highest posterior density (HPD) estimates for each well-supported clade are represented by bars. The APG (2016) classification is indicated to the right of the tree and colored within the tree. Yellow pentagons indicate six calibration points (see text).

Divergence time analyses based on 139 plastomes representing 127 Liliales species and 11 outgroups, with four fossil calibration points and two other calibration points, resulted in an inferred crown-group age of Liliaceae of ∼64.67 Ma (95% HPD: 78.61–51.87 Ma), and the stem age of Liliaceae was estimated as ∼79.87 Ma (95% HPD: 95.66–66.89 Ma, Figure 2 and Table 1). The stem age of Lilieae was estimated as ∼43.04 Ma (95% HPD: 54.07–32.50 Ma), and the age of the crown group was ∼25.16 Ma (95% HPD: 32.60–18.43 Ma, Figure 2 and Table 1). Within Lilieae, the stem age of genus *Notholirion* is predicted to have originated at ∼25.16 Ma (95% HPD: 32.60–18.43 Ma), and the genus *Cardiocrinum* originated at ∼22.89 Ma (95% HPD: 29.73–16.77 Ma). The timing of the divergence between *Lilium* and *Fritillaria* was ∼18.60 Ma (95% HPD: 23.99–13.79 Ma), and then *Lilium* diverged into two lineages (i.e., L1 and L2; Figure 2) at ∼15.79 Ma (95% HPD: 20.87–11.41 Ma). In *Fritillaria*, *F. maximowiczii* stem age was∼18.41 Ma (95% HPD: 23.74–13.68 Ma, Figure 2). We predicted that the tribe Lilieae originated around the Eocene (∼53–36 Ma), with genus and species diversity in the tribe significantly increasing since the Miocene (∼23–5 Ma).

**TABLE 1:** Inferred stem and crown ages (Ma) of Lilieae and other Liliales taxa, with upper and lower bounds of the 95% higher posterior density (HPD) for mean ages based on the analysis with 139 plastomes and six fossils calibration points (four based on fossils) (see Figure 2).

Clade	Stem age	95% HPD	Crown age	95% HPD
Liliaceae	79.87	95.66–66.89	64.67	78.61–51.87
Subfam. Lilioideae	53.57	71.53–37.72	40.04	54.17–24.24
Tribe Lilieae	43.04	54.07–32.50	25.16	32.60–18.43
*Notholirion*	25.16	32.60–18.43	5.65	10.58–1.98
*Cardiocrinum*	22.89	29.73–16.77	6.93	12.84–2.70
*Lilium*	18.60	23.99–13.79	15.79	20.87–11.41
*Fritillaria*	18.60	23.99–13.79	18.41	23.74–13.68
Tribe Tulipeae	43.04	54.07–32.50	34.87	45.78–23.06
Tribe Medeoleae	48.25	59.40–135.99	-	-
Subfam. Tricyrtidoideae	62.96	76.77–50.28	-	-
Subfam. Calochortoideae	64.67	78.61–51.87	57.05	73.12–41.62
Smilacaceae	75.92	38.56–26.23	32.07	41.73–21.65
Philesiaceae	50.96	51.92–49.99	8.09	16.12–1.95
Ripogonaceae	50.96	51.92–49.99	-	-
Melanthiaceae	106.78	120.85–92.54	74.92	95.37–55.44
Colchicaceae	67.05	82.82–53.05	42.16	55.37–29.07
Alstroemeriaceae	67.05	82.82–53.05	23.41	24.45–22.45
Petermanniaceae	97.00	113.15–81.57	-	-
Campynemataceae	122.61	133.53–110.17	-	-

**FIGURE 3:**
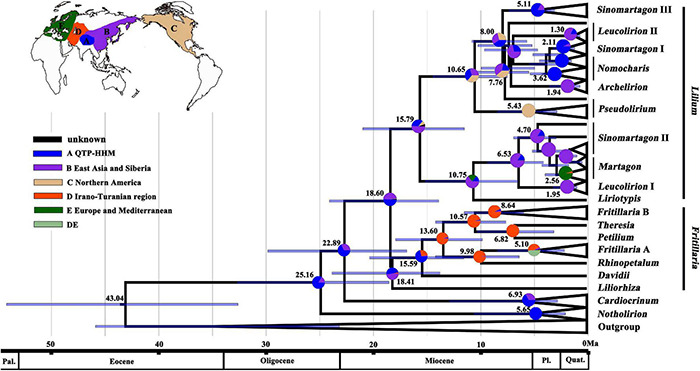
Reconstructions of the ancestral area based on plastome-based phylogeny of Lilieae (see Figure 1A). Pie charts show proportions of inferred ancestral ranges, pie slice colors to regions defined in the caption and inset world map. Mean divergence ages are given on nodes (see Figure 2). Bars on nodes indicate the 95% HPD of divergence ages.

The reconstructions of the ancestral area based on the BEAST plastome analyses (Figure 3) supported the most likely ancestral distribution of Lilieae as being in QTP-HHM. *Notholirion* and *Cardiocrinum* are predicted to have diversified *in situ* there, and the most recent common ancestor (MRCA) of *Lilium* was probably distributed in the QTP-HHM or East Asia. The MRCA of sect. *Pseudolirium* of *Lilium* was inferred to have dispersed from Asia to North America, while the MRCA of other *Lilium* lineages diversified in the QTP-HHM and East Asia. For the largest genus of Lilieae, *Fritillaria*, the QTP-HHM and North Asia are reconstructed as the most likely ancestral ranges. More recent dispersal events were inferred from QTP-HHM to the lrano-Turanian region for *Fritillaria*. The lrano-Turanian region is inferred to be the area of origin and diversification for subg. *Rhinoperalum*, *Theresia*, *Petilium*, and *Fritillaria* clade B in *Fritillaria*.

### Evolution of Lilieae Traits

**FIGURE 4:**
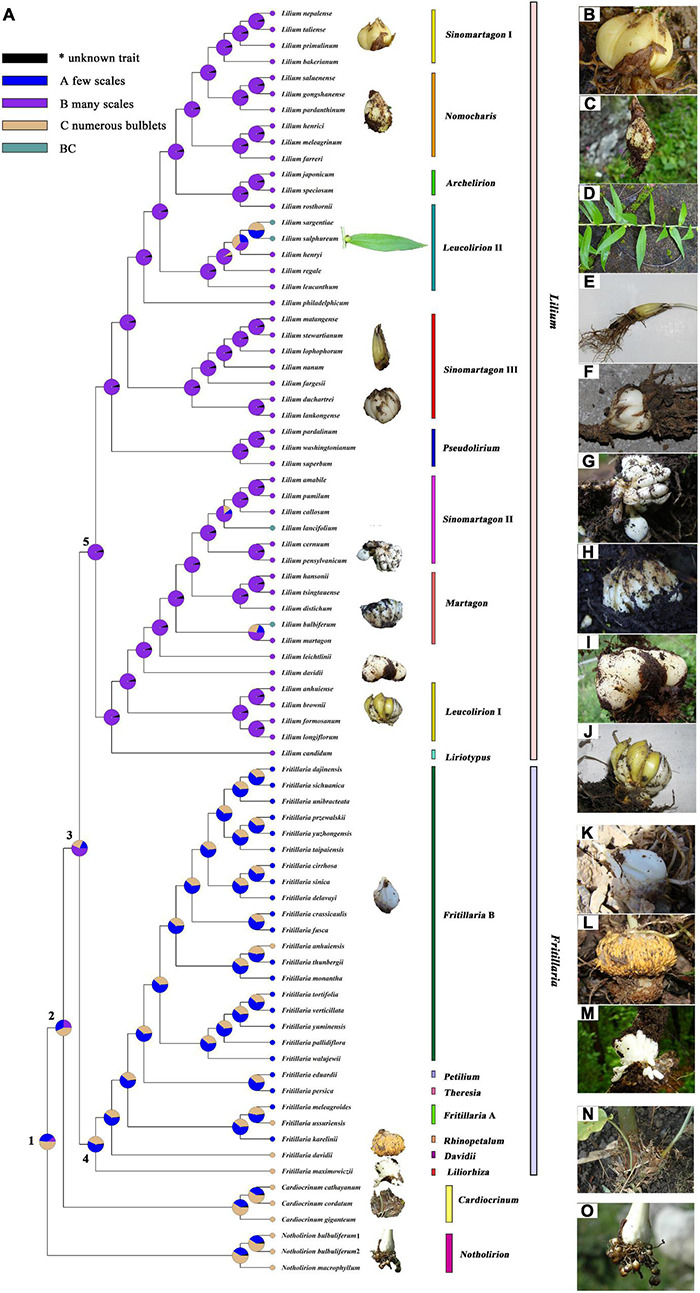
Reconstruction of the ancestral trait of Liliaea bulbs. **(A)** Reconstruction of the ancestral trait; **(B–O)** Bulb types of selected Lilieae species: **(B)** *L. taliense*, **(C)** *L. pardanthinum*, **(D)** *L. sulphureum*, **(E)** *L. lophophorum*, **(F)** *L. duchartrei*, **(G)** *L. pensylvanicum*, **(H)** *L. distichum*, **(I)** *L. davidii*, **(J)** *L. brownii*, **(K)** *F. delavayi*, **(L)** *F. davidii*, **(M)** *F. maximowiczii*, **(N)** *C. giganteum*, and **(O)** *N. bulbuliferum*.

Indices of the phylogenetic signal are shown in Supplementary Table 7 for seven traits in Lilieae. RASP calculated Pagel’s λ (λ; Pagel, 1999), and Blomberg’s *K* value (*K*; Blomberg et al., 2003), which can be used to gage the amount of phylogenetic signal relative to the amount expected for a character undergoing Brownian motion evolution along with the specified topology and branch lengths. The K of bulb component number was 1.08 (λ, 1.04), indicating a good correlation between phylogenetic and bulb component numbers. We inferred a little phylogenetic signal in flower and leaf traits during the diversification of Lilieae (Supplementary Table 7, both K and λ < 1). The reconstruction of an ancestral trait for the bulb type is presented in Figure 4 and Table 2. The results from RASP proposed one possible evolutionary route for Lilieae bulbs. First, the MRCA of Lilieae possibly had numerous bulblets (node 1); second, a phenotype with many scales may have appeared in the MRCA of *Cardiocrinum*, *Fritillaria*, and *Lilium* (node 2). Third, the MRCA of *Fritillaria* and *Lilium* differentiated into bulbs with few scales (node 3). The information for pivotal nodes 1–5 (Figure 4) that represent important ancestors are recorded in Table 2.

**TABLE 2:** Bulb ancestral trait transitions across Lilieae (see Figure 4).

Node	Taxa	Ancestral trait possibility*^1^			Phenotypic evolution event matrix			RASP route*^2^
		A	B	C	Variation	New phenotypes divergence	Extinction	
1	Lilieae most recent common ancestor (MRCA)	37.80	10.58	51.62	0	0	0	C → C ^ C → C| C
2	*Cardiocrinum*, *Fritillaria*, and *Lilium* MRCA	31.91	24.8	43.29	1	1	0	C → CB → C| B
3	*Fritillaria* and *Lilium* MRCA	18.13	56.78	25.10	1	1	0	B → AB → A| B
4	*Fritillaria* MRCA	56.92	2.08	41.00	1	1	0	A → CA → C| A
5	*Lilium* MRCA	1.34	94.80	3.85	0	0	0	B → B ^ B → B| B

*1. A: few scales, B: many scales, C: numerous bulblets.

*2. ^: variation, | : new phenotypes divergence.

## Discussion

### Plastome-Based Inference of Well-Supported Phylogenetic Trees for Lilieae

Our plastome analyses inferred well-supported relationships among genera and species of Lilieae. Among the four Lilieae genera, the monophyly of *Notholirion* and *Cardiocrinum* was reconfirmed, and the uncertain phylogenetic relationships of *Fritillaria* and *Lilium* were resolved (Figure 1). In previous studies (Ronsted et al., 2005; Huang et al., 2018), *Fritillaria* was partitioned into two clades, namely, subgenus *Liliorhiza* (including *F. maximowiczii*) and other subgenera, with *Lilium* nested in two *Fritillaria* clades based on a few plastid sequences. However, *Fritillaria* was monophyletic according to previous ITS-based studies (Hayashi and Kawano, 2000; Ronsted et al., 2005; Lee et al., 2011; Nursel, 2011; Gao et al., 2013; Ghanbari et al., 2018; Huang et al., 2018), which we confirmed here for our ITS-based inferences (Figure 1B). Also, *Fritillaria* is confirmed to be monophyletic (*PP* = 0.82, *BS* = 84) based on the plastome-based tree (Figure 1A). In addition, our plastome analysis also revealed generally well-supported subgeneric relationships in *Fritillaria* and *Lilium*. Relationships among the six subgenera of *Fritillaria* are generally well supported, in line with previous *Fritillaria* studies (Day et al., 2014; Mustafa and Abdul-Razaq, 2015; Mucciarelli et al., 2016; Kiani et al., 2017; Huang et al., 2018). Within *Lilium*, the plastome analyses split the ten sections of this genus into two clades (i.e., L1 and L2) with strong support. Our tree provided a better-supported picture of *Lilium* evolution than many previous analyses (Hayashi and Kawano, 2000; Gao et al., 2013; Du et al., 2014; Ghanbari et al., 2018) and supports a previous plastome study (Givnish et al., 2020). However, the subgeneric clades reconstructed by ITS were weakly supported, especially within *Lilium* (Figure 1B). Nonetheless, our plastome analysis reconstructed a well-supported tree for Lilieae, contributing to a better understanding of Lilieae phylogeny.

### Uplift of the Qinghai-Tibet Plateau Promoted the Divergence Within Lilieae

The Qinghai-Tibet Plateau is one of the important biodiversity hotspots in the world, and climate changes caused its uplifting are thought to have exerted important influences on species evolution in Eurasia (Favre et al., 2015; Renner, 2016; Xing and Ree, 2017; Peterson et al., 2019; Xie et al., 2020b). Even though many details of geological history for the QTP remain controversial, a general consensus has emerged (Wang et al., 2014; Deng and Ding, 2015; Favre et al., 2015; Renner, 2016; Xing and Ree, 2017). For example, the QTP initially uplifted at 45–35 Ma to form a “proto-QTP,” with subsequent extension at 23–15 Ma; uplifts initiated the monsoon system (Wang et al., 2014; Favre et al., 2015; Renner, 2016). By the Middle Miocene, high mountain ranges formed and were accompanied by the aridification of Central Asia (Deng and Ding, 2015; Favre et al., 2015; Renner, 2016). The final extensions of the surrounding mountains, such as the Hengduan Mountains, to current elevations occurred from approximately 10 Ma to the present (Xing and Ree, 2017).

The QTP is an important distribution area and a center of origination and diversity for members of the tribe Lilieae (Gao et al., 2013; Huang et al., 2018; Givnish et al., 2020). Species of Lilieae dispersed in the temperate climatic regions of the northern hemisphere (Boufford, 1993; Chen et al., 2000; Kiani et al., 2017): *Cardiocrinum* species spread over east Asia (Ohara et al., 2006; Yang et al., 2016; Lu et al., 2020) and *Notholirion* species evolved as endemics in the Himalaya mountains (Li et al., 2018, 2020). *Lilium* and *Fritillaria* species are widely distributed in the northern hemisphere, and many *Lilium* species are densely distributed in east Asia (Gao et al., 2013; Gao and Gao, 2016, Gao et al., 2020; Givnish et al., 2016; Liu et al., 2018; Su et al., 2021), whereas species of *Fritillaria* are mainly distributed in central Asia and the Mediterranean region (Wang et al., 2009; Day et al., 2014; Mustafa and Abdul-Razaq, 2015; Mucciarelli et al., 2016). Many species of Lilieae are distributed mainly in the southern and south-eastern regions of the QTP (Wang et al., 2009; Gao et al., 2013; Li et al., 2020), consistent with the QTP being an intensive distribution center of Lilieae species (Chen et al., 2000; Gao and Gao, 2016; Huang et al., 2018; Li et al., 2020). Furthermore, several studies (Gao et al., 2013; Givnish et al., 2020) have concluded that the QTP is a center of origin and diversification for *Lilium*. Other studies have shown that the divergence of *Cardiocrinum* and *Fritillaria* species was promoted by the orogeny of the Hengduan Mountains and QTP, respectively (Yang et al., 2016; Huang et al., 2018). Huang et al. (2018) revealed that the uplift of the QTP and associated climatic changes probably drove early diversification of Lilieae in the QTP region, but this requires verification using age estimates analysis and reconstructions of the ancestral area.

Here, we estimated the timing of the origin of Liliaceae to be around 79.87 Ma (95% HPD: 95.66–66.89 Ma, Figure 2 and Table 1), approximately congruent with several studies, such as 70.428 (44.204–89.435) Ma (Xie et al., 2020a), and 79.8 (59.3–103.0) Ma (Givnish et al., 2016). Divergence time estimates of angiosperms have been heavily influenced by differences in the gene sampling and number and the fossil calibrations used (Foster et al., 2017; Barba-Montoya et al., 2018; Li et al., 2019). Although the divergence time of Lilieae has been estimated previously (Gao et al., 2013; Givnish et al., 2016, 2020; Huang et al., 2018), no study has been conducted on the origin times based on the genome data at the level of the order Liliales. Here, we used 139 plastomes and six calibration points (including four based on fossils) in our age estimates and estimated that Lilieae originated during the Eocene [∼43.04 (54.07–32.50) Ma, Figure 2 and Table 1]. Considering our molecular dating and reconstructions of the ancestral area, this points to several origin and divergence events in Lilieae during the uplifting of the QTP. First, Lilieae may have originated from the QTP-HHM region in the Eocene. Second, a subsequent extension of the QTP and the monsoon system possibly promoted the differentiation between *Cardiocrinum* [∼22.89 (29.73–16.77) Ma] and *Fritillaria* and *Lilium* [∼18.60 (23.99–13.79) Ma]. Third, the pivotal clades and subgenera of *Lilium* and *Fritillaria* diverged from each other in the Middle Miocene. Finally, the diversification of *Lilium* and *Fritillaria* expanded nearly 10 million years ago in the regions surrounding the QTP uplift, which occurred mainly between the Late Miocene and Late Pliocene. Thus, our findings add support to the idea that the uplifting of the QTP promoted the divergence within Lilieae.

### The Most Recent Common Ancestor of Lilieae May Have Had Numerous Bulblets

The bulb is an important taxonomic identifier of Lilieae (Comber, 1949; Boufford, 1993; Chen et al., 2000; Tuyl et al., 2018; Li et al., 2020) and for species reproduction by vegetative propagation (Shoe, 1958; Takayama and Misawa, 1980; Miller and Langhans, 1990; Patterson and Givnish, 2002; Shin et al., 2002; Yan et al., 2019; Givnish et al., 2020). Bulbs are a reliable trait for classification within Lilieae at any time of year, as flowers and leaves are only present for part of the year. Within Lilieae, *Notholirion* species have a bulb with numerous bulblets (Figure 4O), small bulblike organs of vegetative reproduction (Chen et al., 2000; Li et al., 2018, 2020). *Cardiocrinum* species also have bulblets but fewer than *Notholirion* species (Ohara et al., 2006; Yang et al., 2016; Figure 4N). Bulbs of *Lilium* species are composed of many fleshy scales (Chen et al., 2000; Tuyl et al., 2018; Figures 4B–J), and those in *Fritillaria* only possess 1–3 farinaceous scales (Chen et al., 2000; Mucciarelli et al., 2016; Figure 4K). Generally, bulbs of *Lilium* and *Fritillaria* have no bulblets. However, subgenus *Liliorhiza* (including *F. maximowiczii*, Figure 4M) and subgenus *Davidii* (*F. davidii*, Figure 4L) of *Fritillaria* both have bulblets. Previous research revealed that the number of scales per bulb was different in Lilieae and Tulipeae (Patterson and Givnish, 2002; Givnish et al., 2016, 2018). Therefore, to focus on different numbers of storage components of bulbs, we reconstructed the evolution of bulb traits in Lilieae. Figure 4 documents a conceivable evolutionary pathway for the Lilieae bulb. The Lilieae MRCA may have had numerous bulblets, and then bulb type diverged twice in four genera. Furthermore, some species of *Lilium* (e.g., *L. lancifolium*, *L. sulphureum*, *L. sargentiae*, and *L. bulbiferum*) have bulbils (Figure 4D), a type of bulblet growing in leaf axils. This might indicate that bulbs evolved independently at least three times during the *Lilium* diversification. Bulbs with many scales and few scales likely originated after bulbs with bulblets, but this requires verification by increasing taxon sampling and more information on bulb types across taxa.

Bulb evolution in Lilieae may relate to adaptation to the photosynthetic environment during climate oscillation (Patterson and Givnish, 2002; Givnish et al., 2018, 2020). In particular, adaptations related to sexual/asexual reproductive adaptation are important ways for coping with changing environmental conditions in plants (Bengtsson, 2003; Otto and Gerstein, 2006; Silvertown, 2008). As is well known, Lilieae species have both types of reproduction, but each aspect may be emphasized differently across the species (Comber, 1949; Shoe, 1958; Takayama and Misawa, 1980; Tuyl et al., 2018). For example, taxa, such as *N. bulbuliferum*, *L. lancifolium*, and *F. davidii* frequently undergo asexual reproduction using bulblets (Chen et al., 2000; Ohara et al., 2006; Luo et al., 2012; Li et al., 2020). However, bulbs with scales generally provide more nutrients for the organism to generate seeds, and so this form appears to be more focused on sexual reproduction. Because clones are less genetically diverse, they are more vulnerable to habitat disturbance, and so asexual reproduction may be less advantageous during prolonged environmental upheaval (Silvertown, 2008). Sexual reproduction and its potential for recombination should be advantageous when the environment is undergoing perturbation (Baker, 1959; Bender et al., 1984; Bengtsson, 2003; Otto and Gerstein, 2006). Our analysis provides new insights into the evolutionary history of lilies and patterns of QTP species evolution. Significant uplift of the QTP is thought to have greatly intensified the seasonality of monsoonal Asia and initiated a general global decrease in temperature and increase in thermal and moisture seasonality at higher latitudes (Raymo and Ruddiman, 1992; Graham, 1999; Patterson and Givnish, 2002). Combined with the above age estimates, bulb scales might have arisen to enhance sexual reproduction in the MRCA of *Fritillaria* and *Lilium* during the Miocene, allowing adaptation to climatic oscillations due to the QTP uplift.

## Data Availability Statement

The original contributions presented in the study are publicly available. This data can be found at NCBI under accession PRJNA784653.

## Author Contributions

JL, S-DZ, and X-JH conceived the study. JL composed the article. JC and H-HQ performed experiments. YY and ZZ performed the data analysis. D-FX, MP, and X-FG revised the manuscript. All authors read and approved the final manuscript.

## Conflict of Interest

The authors declare that the research was conducted in the absence of any commercial or financial relationships that could be construed as a potential conflict of interest.

## Publisher’s Note

All claims expressed in this article are solely those of the authors and do not necessarily represent those of their affiliated organizations, or those of the publisher, the editors and the reviewers. Any product that may be evaluated in this article, or claim that may be made by its manufacturer, is not guaranteed or endorsed by the publisher.

## Supplementary Material

Supplementary Figure 1 Phylogenetic tree resulting from a maximum likelihood (ML) analysis based on the Lilieae whole plastome (WP) matrix.

Supplementary Figure 2 Phylogenetic tree resulting from an ML analysis based on the Lilieae plastid coding sequences (CDSs) matrix.

Supplementary Figure 3 Phylogenetic tree resulting from a Bayesian inference (BI) analysis based on the Lilieae plastid CDSs matrix.

Supplementary Figure 4 Phylogenetic tree resulting from an ML analysis based on Lilieae nuclear ITS data.
