# Supplementary figures and images for: Phylogeny, Age, and Evolution of Tribe Lilieae (Liliaceae) Based on Whole Plastid Genomes
Source: Front Plant Sci. 2022 Feb 1;12:699226. doi: 10.3389/fpls.2021.699226 (PMC8845482; doi:10.3389/fpls.2021.699226)

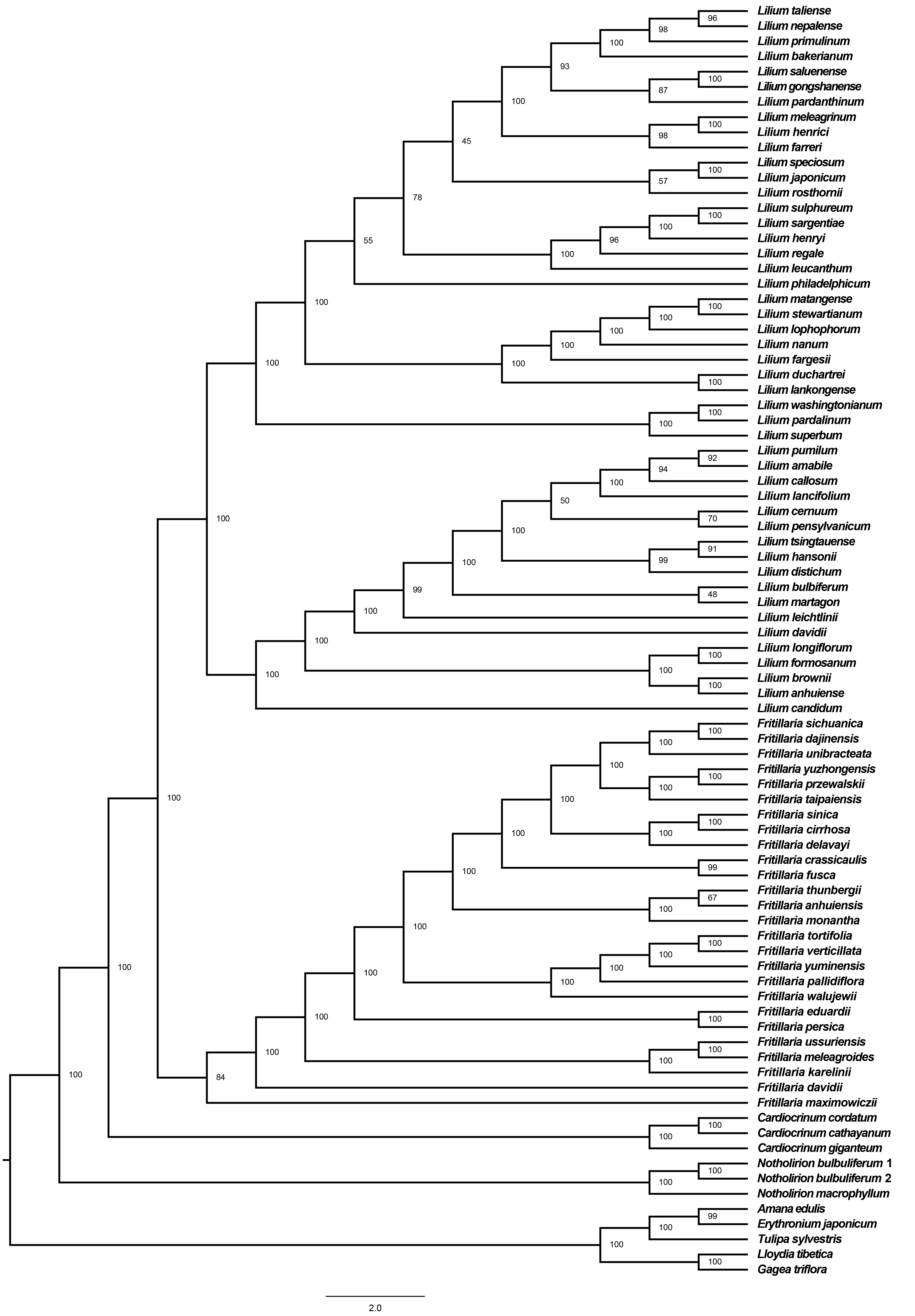

Supplement: Supplementary Figure 1 — Phylogenetic tree resulting from a maximum likelihood (ML) analysis based on the Lilieae whole plastome (WP) matrix. [file Image_1.JPEG]

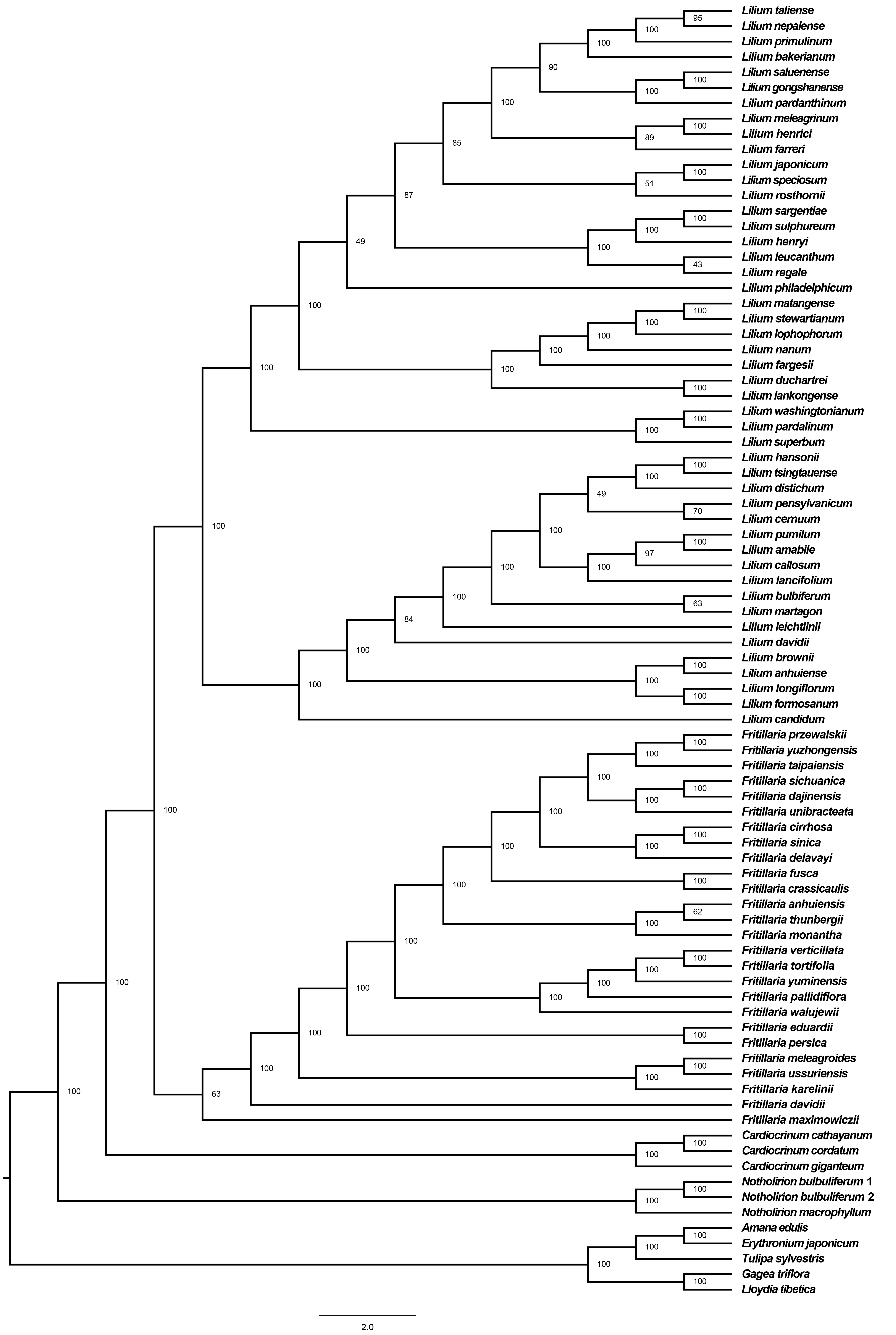

Supplement: Supplementary Figure 2 — Phylogenetic tree resulting from an ML analysis based on the Lilieae plastid coding sequences (CDSs) matrix. [file Image_2.JPEG]

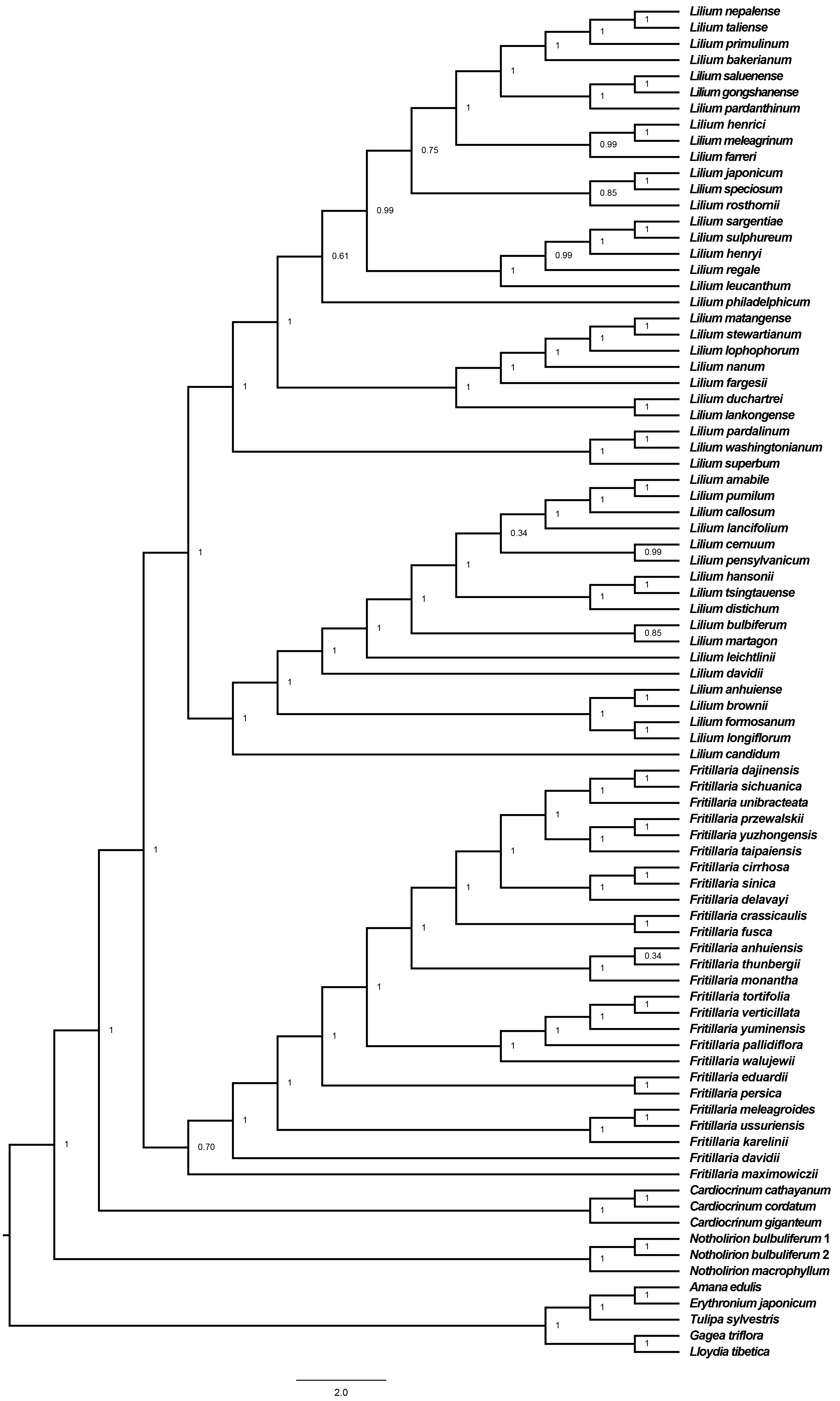

Supplement: Supplementary Figure 3 — Phylogenetic tree resulting from a Bayesian inference (BI) analysis based on the Lilieae plastid CDSs matrix. [file Image_3.JPEG]

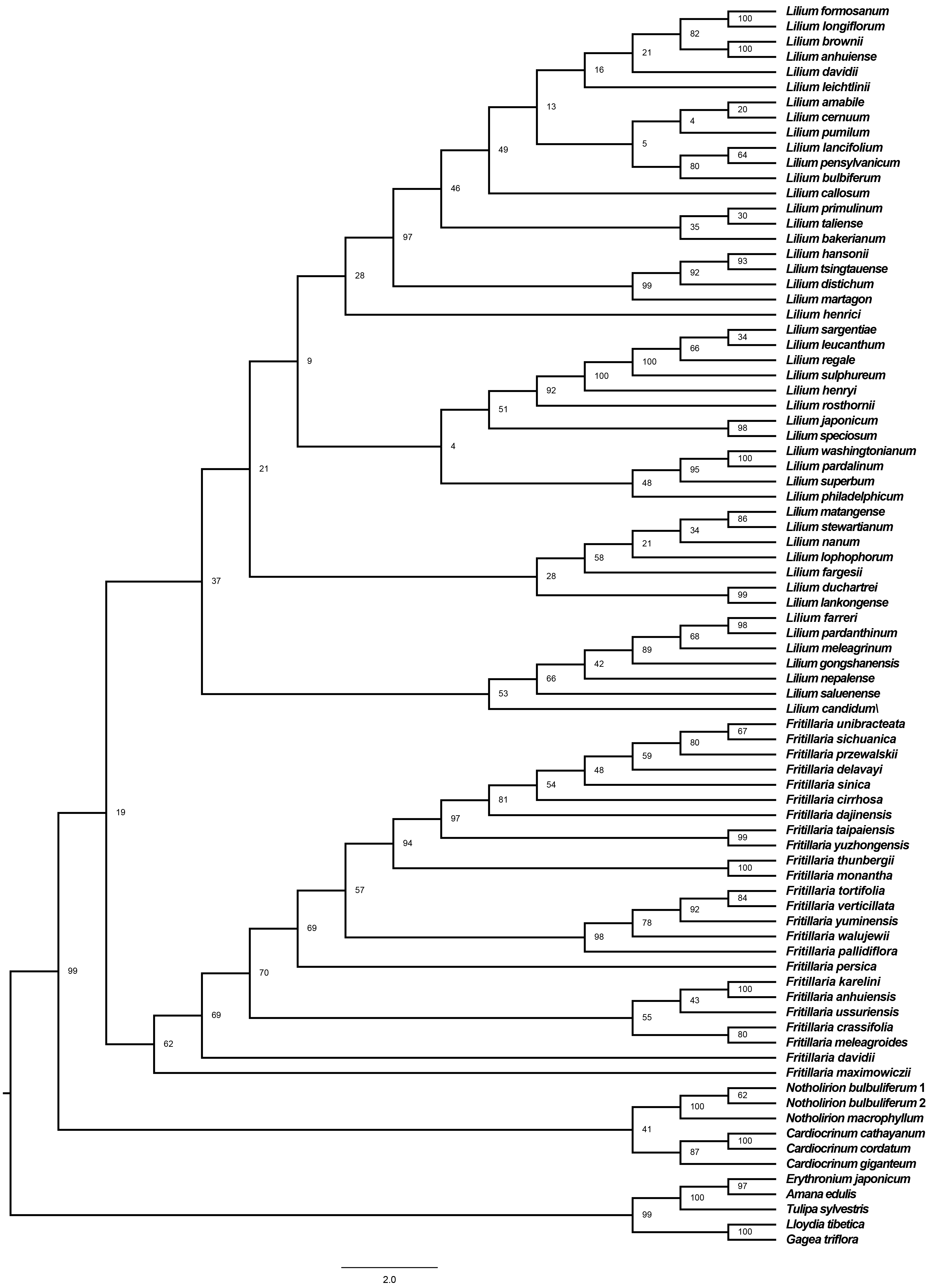

Supplement: Supplementary Figure 4 — Phylogenetic tree resulting from an ML analysis based on Lilieae nuclear ITS data. [file Image_4.JPEG]
