# Supplementary material for: Phylogeny, Age, and Evolution of Tribe Lilieae (Liliaceae) Based on Whole Plastid Genomes
Source: Front Plant Sci. 2022 Feb 1;12:699226. doi: 10.3389/fpls.2021.699226 (PMC8845482; doi:10.3389/fpls.2021.699226)
Supplement: Supplementary file 5 [file Table_1.DOCX]

**Supplementary table 1 |** List of species included in this study, information including GenBank accession numbers, natural distribution details, and information on sample collection.

| Organism | Plastome Accession | ITS Accession | Natural distribution^*1^ | Area^*2^ | Voucher (SZ) | Sampling locality |
| --- | --- | --- | --- | --- | --- | --- |
| **Liliales-Liliaceae** | | | | | | |
| the tribe Lilieae | | | | | | |
| *Fritillaria anhuiensis* | MK258148 | MH588430 | E. China | B | H20160415 | Yuexi, Anhui province |
| *Fritillaria crassicaulis* | MK258147 | MF083540 | SC. China | A | H20160518 | Zhongdian, Yunnan province |
| *Fritillaria dajinensis* | MK258146 | MF083547 | SC. China | A | H20160628 | Jinchuan, Sichuan province |
| *Fritillaria davidii* | MK258145 | MF083554 | W. China (W. Sichuan) | A | H20170407 | Baoxing, Sichuan province |
| *Fritillaria delavayi* | MK258144 | MF083557 | SC. China to Sikkim | A | G20100903 | Zhongdian, Yunnan province |
| *Fritillaria fusca* | MW890003 | ———— | SC. China | A | XZ10219 (PE) | Dengmu, Xizang Autonomous Region |
| *Fritillaria maximowiczii* | MK258138 | MG525328 | N. China and E. Siberia | B | X20170510 | Jiamusi, Heilongjiang province |
| *Fritillaria monantha* | MK258143 | MF083559 | C. China | B | H20160409 | Anji, Zhejiang province |
| *Fritillaria przewalskii* | MK258142 | MF083551 | SC. China | A | H20160610 | Zhangxian, Gansu province |
| *Fritillaria sichuanica* | MK258141 | MF083553 | SC. China | A | H20160626 | Baoxing, Sichuan province |
| *Fritillaria unibracteata* | MK258140 | MF083535 | C. China | B | H20160701 | Lixian, Sichuan province |
| *Fritillaria yuzhongensis* | MK258139 | MF083562 | NC. China, Inner Mongolia | B | H20160607 | Yuzhong, Gansu province |
| *Fritillaria cirrhosa* | MH244906 | MF083539 | Himalaya to C. China | A | ———— | ———— |
| *Fritillaria karelinii* | KX354691 | AY616742 | C. Asia to NW. China | D | ———— | ———— |
| *Fritillaria eduardii* | NC037038 | ———— | Central Asia | D | ———— | ———— |
| *Fritillaria meleagroides* | NC037040 | MG946142 | WC. Bulgaria to NW. China | DE | ———— | ———— |
| *Fritillaria pallidiflora* | NC037216 | AY616735 | C. Asia to NW. China | D | ———— | ———— |
| *Fritillaria persica* | NC037039 | AY616736 | S. Turkey to Iran and Israel | DE | ———— | ———— |
| *Fritillaria sinica* | NC044631 | KP906211 | SC. China | A | ———— | ———— |
| *Fritillaria taipaiensis* | MH244910 | MF083558 | C.China | B | ———— | ———— |
| *Fritillaria thunbergii* | NC034368 | MF083548 | E. China | B | ———— | ———— |
| *Fritillaria tortifolia* | NC037214 | KP712004 | NW. China | D | ———— | ———— |
| *Fritillaria ussuriensis* | NC034369 | DQ191622 | Primorye to Korea | B | ———— | ———— |
| *Fritillaria verticillata* | NC037217 | AY616735 | NW. China to W.Siberia | D | ———— | ———— |
| *Fritillaria walujewii* | NC037215 | KP712008 | NW. China to C. Asia | D | ———— | ———— |
| *Fritillaria yuminensis* | NC037209 | HM045472 | NW. China | D | ———— | ———— |
| *Lilium anhuiense* | MW890005 | HM045454 | E. China | B | G2009016 | Shitaixian, Anhui province |
| *Lilium brownii* | MK493294 | HM045448 | China | B | ZZS2017062311 | Dujiangyan, Sichuan province |
| *Lilium davidii* | MW890008 | HM045426 | SC. China | A | LJ2017062310 | Jinchuan, Sichuan province |
| *Lilium distichum* | MK493296 | HM045451 | NE.China to Russia | B | WCB71325 | Huoshankou, Heilongjiang province |
| *Lilium duchartrei* | MN745202 | HM045429 | SC. & C. China | A | KS20180630 | Kangding, Sichuan province |
| *Lilium farreri* | MW890004 | HM045437 | SC. China to N. Myanmar | A | LS20180724 | Lushui, Yunnan province |
| *Lilium gongshanense* | MK493297 | KP711995 | SC. China | A | LJ2017072322 | Gongshan, Yunnan province |
| *Lilium henrici* | NC039436 | HM045456 | SC. China | A | LJ2017072513 | Tengchong, Yunnan province |
| *Lilium lankongense* | MK757466 | HM045430 | SC. China | A | KS20180716 | Lijiang, Yunnan province |
| *Lilium lophophorum* | MK493298 | KF851105 | SC. China | A | LJ2017062301 | Jinchuan, Sichuan province |
| *Lilium matangense* | MN745201 | HM045457 | SC. China | A | LS20180622 | Matang, Sichuan province |
| *Lilium meleagrinum* | MK493299 | HM045436 | SC. China | A | G20090711 | Gongshan, Yunnan province |
| *Lilium nepalense* | MK493301 | AB020444 | Himalaya to SW. China | A | LJ2017072502 | Tengchong, Yunnan province |
| *Lilium pardanthinum* | NC038193 | HM045434 | SC. China | A | LJ2017072315 | Gongshan, Yunnan province |
| *Lilium pensylvanicum* | MK493295 | HM045446 | China, Korea, Japan, Russia | B | SMZ20170711 | Paektu Mountain, Jilin province |
| *Lilium rosthornii* | MW890009 | KF851106 | SW. China | B | L2014081603 | Wulong, Chongqing province |
| *Lilium saluenense* | MK493293 | HM045434 | SW. China to N. Myanmar | A | LJ2017072305 | Gongshan, Yunnan province |
| *Lilium stewartianum* | MN745202 | HQ692154 | SC.China | A | SDM20180702 | Xiangcheng, Sichuan province |
| *Lilium sulphureum* | MK493304 | HM045464 | SW. China to Myanmar | B | G20090714-1-1 | Xichou, Yunnan province |
| *Lilium nanum* | MK493300 | HM045458 | Himalaya to SW. China | A | YM20140828 | Linzhi, Xizang Autonomous Region |
| *Lilium regale* | MK493302 | HQ456831 | SC. China | A | LJ2017062311 | Jinchuan, Sichuan province |
| *Lilium sargentiae* | MK493303 | HQ456832 | SW. China | B | LHY2017071302 | Yibing, Sichuan province |
| *Lilium speciosum var. gloriosoides* | MW890010 | HM045461 | E. China | B | JQP18082301 | Baima Mountain, Zhejiang province |
| *Lilium amabile* | NC035988 | HQ456828 | NE. China, Korea | B | ———— | ———— |
| *Lilium bakerianum* | NC035592 | HM045428 | SW. China, Nepal to N. Myanmar | A | ———— | ———— |
| *Lilium bulbiferum* | NC037517 | AF090952 | C. Europe to S. Italy | E | ———— | ———— |
| *Lilium callosum* | NC035989 | KJ710108 | S. China to Japan | B | ———— | ———— |
| *Lilium candidum* | NC042399 | AF092522 | S. Macedonia to SW. Turkey | E | ———— | ———— |
| *Lilium cernuum* | NC034840 | HM045427 | NE. China, Korea, Russia | B | ———— | ———— |
| *Lilium fargesii* | NC033908 | HM045459 | SW. & C. China | B | ———— | ———— |
| *Lilium formosanum* | NC042398 | AB020470 | China (TaiWan) | B | ———— | ———— |
| *Lilium hansonii* | NC027674 | AB020448 | Korea | B | ———— | ———— |
| *Lilium henryi* | NC035570 | HM045462 | SW. China | B | ———— | ———— |
| *Lilium japonicum* | MT261164 | AF074471 | Japan | B | ———— | ———— |
| *Lilium lancifolium* | NC035589 | HM045465 | China, Japan, Russian Far East | B | ———— | ———— |
| *Lilium leichtlinii var. maximowiczii* | MK753242 | HQ686072 | China, Korea, Japan, Russian | B | ———— | ———— |
| *Lilium leucanthum* | NC035590 | HM045463 | C. China | B | ———— | ———— |
| *Lilium longiflorum* | KC968977 | HM045447 | Japan | B | ———— | ———— |
| *Lilium martagon var. pilosiusculum* | NC039162 | HM045452 | NW. China to Europe | DE | ———— | ———— |
| *Lilium pardalinum* | MH029495 | AB020439 | Oregon to Mexico | C | ———— | ———— |
| *Lilium philadelphicum* | NC035990 | AB020432 | Canada to WC. & E. U.S.A. | C | ———— | ———— |
| *Lilium primulinum var. ochraceum* | KY748298 | HM045450 | SW. China | B | ———— | ———— |
| *Lilium pumilum* | MN906760 | HM045445 | China, Korea, Russian | B | ———— | ———— |
| *Lilium superbum* | NC026787 | AF090960 | EC. & E. U.S.A. | C | ———— | ———— |
| *Lilium taliense* | NC034370 | HM045425 | SW. China | A | ———— | ———— |
| *Lilium tsingtauense* | KU230438 | HQ687259 | E. China to Korea | B | ———— | ———— |
| *Lilium washingtonianum* | NC037699 | AM162674 | W. Oregon to NC. California | C | ———— | ———— |
| *Notholirion bulbuliferum*1 | MW890007 | HQ448856 | SC. China to Nepal | A | JQL0608 | Meixian, Shanxi province |
| *Notholirion bulbuliferum*2 | MW890006 | KP712026 | SC. China to Nepal | A | ZJGS0802 | Gongshan, Yunnan province |
| *Notholirion macrophyllum* | MH011354 | HM045475 | SC. China to Nepal | A | LJ2017080601 | Daocheng, Sichuan province |
| *Notholirion thomsonianum* | MZ128661 | ———— | Afghanistan to Nepal | A | LJ202102 | Southern Nepal |
| *Cardiocrinum giganteum* | NC033896 | HM045473 | SC.China to NW. Myanmar | A | ———— | ———— |
| *Cardiocrinum cathayanum* | NC033897 | HM045474 | Sakhalin, S. Kuril Is., Japan | B | ———— | ———— |
| *Cardiocrinum cordatum* | NC033898 | KP712019 | C. China | B | ———— | ———— |
| the tribe Tulipeae | | | | | | |
| *Erythronium japonicum* | MT261155 | EU912083 | ———— | —— | ———— | ———— |
| *Erythronium sibiricum* | NC035681 | ———— | ———— | —— | ———— | ———— |
| *Amana erythronioides* | NC034634 | ———— | ———— | —— | ———— | ———— |
| *Amana wanzhensis* | NC034705 | ———— | ———— | —— | ———— | ———— |
| *Amana anhuiensis* | NC034706 | ———— | ———— | —— | ———— | ———— |
| *Amana edulis* | NC034707 | KP712031 | ———— | —— | ———— | ———— |
| *Amana kuocangshanica* | NC034708 | ———— | ———— | —— | ———— | ———— |
| *Tulipa altaica* | MW077741 | ———— | ———— | —— | LJ20190515 | Yuming, Xinjiang Uygur Autonomous Region |
| *Tulipa iliensis* | MW077740 | ———— | ———— | —— | LJ20190516 | Yuming, Xinjiang Uygur Autonomous Region |
| *Tulipa patens* | MW077739 | ———— | ———— | —— | LJ20190515-2 | Yuming, Xinjiang Uygur Autonomous Region |
| *Tulipa thianschanica* | MW077738 | ———— | ———— | —— | LJ20190517 | Yuming, Xinjiang Uygur Autonomous Region |
| *Tulipa sylvestris* | MT261172 | JQ776491 | ———— | —— | ———— | ———— |
| *Gagea triflora* | MT261157 | AM162674 | ———— | —— | ———— | ———— |
| *Lloydia tibetica* | MW890011 | KP712029 | ———— | —— | ———— | ———— |
| the tribe Medeoleae | | | | | | |
| *Clintonia udensis* | MT261153 | ———— | ———— | —— | ———— | ———— |
| subfamlify Tricyrtidoideae | | | | | | |
| *Streptopus ovalis* | MT261171 | ———— | ———— | —— | ———— | ———— |
| *Tricyrtis macropoda* | NC040223 | ———— | ———— | —— | ———— | ———— |
| Subfamlify Calochortoideae | | | | | | |
| *Calochortus venustus* | MT261150 | ———— | ———— | —— | ———— | ———— |
| **Liliales-Smilacaceae** | | | | | | |
| *Smilax* sp. | MW890012 | ———— | ———— | —— | LJ2019061002 | Emei mountain, Sichuan province |
| *Smilax china* | HM536959 | ———— | ———— | —— | ———— | ———— |
| *Smilax glycophylla* | NC049023 | ———— | ———— | —— | ———— | ———— |
| *Smilax nipponica* | NC049024 | ———— | ———— | —— | ———— | ———— |
| **Liliales-Ripogonaceae** | | | | | | |
| *Ripogonum scandens* | MT261167 | ———— | ———— | —— | ———— | ———— |
| **Liliales-Philesiaceae** | | | | | | |
| *Lapageria rosea* | NC049017 | ———— | ———— | —— | ———— | ———— |
| *Philesia magellanica* | MT261166 | ———— | ———— | —— | ———— | ———— |
| **Liliales-Colchicaceae** | | | | | | |
| *Uvularia grandiflora* | NC049026 | ———— | ———— | —— | ———— | ———— |
| *Disporum sessile* | NC045518 | ———— | ———— | —— | ———— | ———— |
| *Tripladenia cunninghamii* | NC049025 | ———— | ———— | —— | ———— | ———— |
| *Wurmbea burttii* | NC049027 | ———— | ———— | —— | ———— | ———— |
| *Gloriosa superba* | NC030065 | ———— | ———— | —— | ———— | ———— |
| *Colchicum autumnale* | NC030064 | ———— | ———— | —— | ———— | ———— |
| *Androcymbium greuterocymbium* | MT261148 | ———— | ———— | —— | ———— | ———— |
| **Liliales-Alstroemeriaceae** | | | | | | |
| *Luzuriaga radicans* | NC025333 | ———— | ———— | —— | ———— | ———— |
| *Bomarea edulis* | NC025306 | ———— | ———— | —— | ———— | ———— |
| *Alstroemeria aurea* | KC968976 | ———— | ———— | —— | ———— | ———— |
| *Alstroemeria hybrid* | NC041554 | ———— | ———— | —— | ———— | ———— |
| **Liliales-Petermanniaceae** | | | | | | |
| *Petermannia cirrosa* | MT261165 | ———— | ———— | —— | ———— | ———— |
| **Liliales-Melanthiaceae** | | | | | | |
| *Veratrum patulum* | KF437397 | ———— | ———— | —— | ———— | ———— |
| *Chionographis japonica* | KF951065 | ———— | ———— | —— | ———— | ———— |
| *Heloniopsis tubiflora* | KM078036 | ———— | ———— | —— | ———— | ———— |
| *Xerophyllum tenax* | KM078035 | ———— | ———— | —— | ———— | ———— |
| *Ypsilandra thibetica* | MH796671 | ———— | ———— | —— | ———— | ———— |
| *Trillium camschatcense* | MN125568 | ———— | ———— | —— | ———— | ———— |
| *Paris dunniana* | KX784042 | ———— | ———— | —— | ———— | ———— |
| *Paris birmanica* | MN125571 | ———— | ———— | —— | ———— | ———— |
| *Paris bashanensis* | MN125580 | ———— | ———— | —— | ———— | ———— |
| *Paris axialis* | MN125591 | ———— | ———— | —— | ———— | ———— |
| **Liliales-Campynemataceae** | | | | | | |
| *Campynema lineare* | NC026785 | ———— | ———— | —— | ———— | ———— |
| **Outgroups** | | | | | | |
| *Areca catechu* | MT559306 | ———— | ———— | —— | ———— | ———— |
| *Asparagus setaceus* | MT712152 | ———— | ———— | —— | ———— | ———— |
| *Canna indica* | KF601570 | ———— | ———— | —— | ———— | ———— |
| *Costus viridis* | MK262733 | ———— | ———— | —— | ———— | ———— |
| *Heliconia collinsiana* | JX088660 | ———— | ———— | —— | ———— | ———— |
| *Ravenala madagascariensis* | KF601568 | ———— | ———— | —— | ———— | ———— |
| *Roscoea alpina* | NC050946 | ———— | ———— | —— | ———— | ———— |
| *Sparganium stoloniferum* | MK460210 | ———— | ———— | —— | ———— | ———— |
| *Thaumatococcus daniellii* | KF601575 | ———— | ———— | —— | ———— | ———— |
| *Xiphidium caeruleum* | JX088669 | ———— | ———— | —— | ———— | ———— |
| *Zingiber mioga* | MW067010 | ———— | ———— | —— | ———— | ———— |

*1 The natural distributions of the taxa were obtained from the WCSP 2014 (http://wcsp.science.kew.org).

*2. A, the Qinghai Tibet Plateau-Himalayas-Hengduan Mountains (QTP-HHM); B, East Asia and Siberia; C, Northern America; D, Irano-Turanian region (central and western Asia, north-east Africa and north-west China); E, Europe and Mediterranean Basin.
