# Supplementary material for: Phylogeny, Age, and Evolution of Tribe Lilieae (Liliaceae) Based on Whole Plastid Genomes
Source: Front Plant Sci. 2022 Feb 1;12:699226. doi: 10.3389/fpls.2021.699226 (PMC8845482; doi:10.3389/fpls.2021.699226)
Supplement: Supplementary file 6 [file Table_2.DOCX]

**Supplementary table 2 |** Partitions and models for Lilieae phylogenetic analysis.

| Sequences in partition | Best model | Site positions in data matrix |
| --- | --- | --- |
| rrn4.5, trnF_GAA, trnI_CAU, trnL_CAA, trnM_CAU, trnR_UCU, trnT_GGU, trnV_GAC | GTR | 1-103, 71156-71228, 72175-72248, 76171-76251, 77011-77082, 77375-77447, 77718-77789, 77863-77934 |
| clpP, rpl16, rpl22, rps19, trnL_UAA | GTR+I | 9094-11259, 43043-44722, 45077-45469, 62493-62771, 76252-76930 |
| atpB-rbcL, clpP-psbB, matK | GTR+I | 95206-95871, 97788-98318, 11260-12822 |
| ndhB, psbF | GTR | 15085-17304, 39814-39933 |
| ndhB-rps7, ndhC, psaI, psaJ, psbL, psbT | GTR+I | 100171-100484, 17305-17667, 34261-34365, 34366-34494, 40582-40698, 40940-41041 |
| ndhD, ndhE, petL | GTR+G | 17668-19176, 19177-19482, 29371-29466 |
| ndhF | GTR+I | 19483-21765 |
| ndhG, psbI, psbM | GTR+G | 21766-22338, 40156-40266, 40699-40807 |
| ndhH, rpl14, rpoC2 | GTR+I | 22339-23520, 42674-43042, 54871-59055 |
| ndhI, rpl20-rps12 | GTR+I | 23521-24060, 109072-109919 |
| ndhJ, trnN-ndhF | GTR+I | 24061-24537, 121002-122565 |
| accD, rpl32, rpl33, rps15 | GTR+G | 104-1585, 47274-47447, 47448-47651, 60702-60974 |
| ndhK, rps18, rps4 | GTR+I | 24538-25440, 62187-62492, 64140-64745 |
| petD, rpl20, trnV_UAC | GTR+I | 27957-29256, 44723-45076, 77935-78624 |
| psaA, psaB | GTR+I | 29557-31809, 31810-34014 |
| psaC, psbE, rpl36 | GTR | 34015-34260, 39562-39813, 47652-47765 |
| psbK, psbZ | GTR+G | 40390-40581, 41042-41230 |
| rbcL | GTR+I | 41231-42673 |
| rpl2, rpl23, rps12, rps7, trnV-rrn16 | GTR+I | 45752-47273, 45470-45751, 59479-60398, 64746-65213, 127221-127447 |
| rpoA, rps3, rps8 | GTR+G | 47766-48776, 63483-64139, 65214-65612 |
| rpoC1, trnS_UGA, ycf3 | GTR+I | 51984-54870, 77625-77717, 91226-93294 |
| psaI-ycf4, rps16, ycf3-trnS | GTR+I | 103804-104182, 60975-62186, 128520-129127 |
| atpA, atpB, petA, psbH, psbN, rps11 | GTR+I | 1586-3109, 3110-4612, 25441-26403, 39934-40155, 40808-40939, 59056-59478 |
| rrn16, rrn23 | GTR+I | 65613-67103, 67104-69914 |
| rrn5, trnA_UGC, trnC_GCA, trnI_GAU, trnW_CCA | GTR+I | 69915-70035, 70036-70927, 70928-71008, 72249-73260, 78625-78698 |
| trnD_GUC, trnE_UUC, trnfM_CAU, trnG_UCC, trnH_GUG, trnL_UAG, trnN_GUU, trnP_UGG, trnQ_UUG, trnR_ACG, trnS_GGA | GTR+I | 71009-71082, 71083-71155, 78783-78856, 72029-72099, 72100-72174, 76931-77010, 77083-77154, 77155-77228, 772 |
| psbE-petL, trnK_UUU | GTR+I | 105095-106453, 73261-76170 |
| ycf1 | GTR+I | 78857-84505 |
| trnL-ndhB, ycf2 | GTR+I | 120411-121001, 84506-91225 |
| rrn4.5-rrn5 | GTR+I | 93850-94086 |
| accD_psaI | GTR+G | 94087-95205 |
| atpF-atpH, ndhC-trnV, rpl36-rps8, ycf4-cemA | GTR+G | 95872-96469, 100485-101305, 110802-111393, 129128-129974 |
| atpH-atpI, psbM-trnD, rpoB-trnC | GTR+I | 96470-97512, 106454-107866, 111394-112245 |
| atpE, rpoB, rps14, rps2, trnR-trnN | GTR+G | 4613-5021, 48777-51983, 60399-60701, 62772-63482, 123327-123849 |
| atpI_rps2 | GTR+G | 97513-97787 |
| matK-rps16, psbZ-trnG, trnD-trnY | GTR+I | 98319-100170, 107867-108218, 118321-118706 |
| ndhF-rpl32, rps16-trnQ | GTR+I | 101306-101851, 112713-113613 |
| petA-psbJ, trnC-petN | GTR+I | 101852-102725, 117692-118320 |
| psaA-ycf3 | GTR+I | 102726-103394 |
| psaC-ndhE, trnE-trnT, trnS-trnG | GTR+I | 103395-103803, 118707-119561, 123850-124823 |
| psaJ-rpl33, trnT-trnL | GTR+I | 104183-104825, 126138-127220 |
| psbA-trnK | GTR | 104826-105094 |
| rbcL-accD | GTR+I | 108219-109071 |
| rpl32-trnL, rps15-ycf1 | GTR+I | 109920-110801, 112246-112712 |
| atpF | GTR+I | 5022-6435 |
| rps18-rpl20, trnF-ndhJ | GTR+G | 113614-113921, 119562-120410 |
| rps2-rpoC2 | GTR+G | 113922-114235 |
| rps4-trnT | GTR+I | 114236-114600 |
| rps7-trnV | GTR+G | 114601-117378 |
| rrn16-trnI | GTR+I | 117379-117691 |
| trnP-psaJ, trnQ-psbK | GTR+I | 122566-122974, 122975-123326 |
| trnT-psbD | GTR+I | 124824-126137 |
| ycf2-trnL | GTR+I | 127448-128519 |
| atpH, petN, psbB, psbC, psbD, psbJ, trnS_GCU | GTR+I | 6436-6681, 29467-29556, 35557-37083, 37084-38499, 38500-39561, 40267-40389, 77448-77537 |
| atpI, petG, psbA, ycf4 | GTR+I | 6682-7425, 29257-29370, 34495-35556, 93295-93849 |
| ccsA, ndhA, petB, trnG_GCC | GTR+I | 7426-8397, 12823-15084, 26404-27956, 71229-72028 |
| cemA | GTR+I | 8398-9093 |
