# Supplementary material for: Phylogeny, Age, and Evolution of Tribe Lilieae (Liliaceae) Based on Whole Plastid Genomes
Source: Front Plant Sci. 2022 Feb 1;12:699226. doi: 10.3389/fpls.2021.699226 (PMC8845482; doi:10.3389/fpls.2021.699226)
Supplement: Supplementary file 7 [file Table_3.DOCX]

**Supplementary table 3 |** Partitions and models for divergence time estimation of Lilieae.

| Sequences in partition | Best model | Site positions in data matrix |
| --- | --- | --- |
| accD | GTR+I | 1-1604 |
| atpA, atpB | GTR+I | 1605-3128, 3129-4634 |
| atpE, rpl14, rpoB, rps14, rps18, rps4 | GTR+I | 4635-5074, 37198-37568, 41451-44726, 51985-52298, 52584-52916, 54612-55223 |
| atpF, ycf, | GTR+I | 5075-5635, 71113-71667 |
| atpH, petN, psbE, psbF, psbJ | GTR+I | 5636-5881, 23868-23963, 33982-34233, 34234-34353, 34704-34826 |
| atpI, psaI, psbI, psbM, psbZ | GTR+I | 5882-6625, 28668-28778, 34588-34703, 35136-35240, 35492-35686 |
| ccsA | GTR+G | 6626-7652 |
| cemA | GTR+I | 7653-8354 |
| clpP, ndhK | GTR+I | 8355-8987, 20604-21538 |
| matK | GTR+I | 8988-10577 |
| ndhA, ndhE | GTR+G | 10578-11727, 15145-15455 |
| ndhB | GTR+I | 11728-13260 |
| ndhC, psbD | GTR+I | 13261-13623, 32919-33981 |
| ndhD | GTR+G | 13624-15144 |
| ndhF | GTR+G | 15456-17833 |
| ndhG | GTR+G | 17834-18365 |
| ndhH, psaC | GTR+G | 18366-19572, 28422-28667 |
| ndhI | GTR+G | 19573-20123 |
| ndhJ, psbH | GTR+I | 20124-20603, 34354-34587 |
| petA, rpl36, rpoC1, rps2 | GTR+I | 21539-22501, 40267-40380, 44727-46866, 53202-53912 |
| petB, petD, psaB | GTR+I | 22502-23149, 23150-23652, 26217-28421 |
| petG, petL | GTR+G | 23653-23766, 23767-23867 |
| psaA, psbC, psbN | GTR+I | 23964-26216, 31497-32918, 35241-35377 |
| psaJ, psbK | GTR+I | 28779-28907, 34827-35018 |
| psbA | GTR+I | 28908-29969 |
| psbB | GTR+I | 29970-31496 |
| psbL | GTR+G | 35019-35135 |
| psbT | GTR+I | 35378-35491 |
| rbcL | GTR+I | 35687-37197 |
| rpl16, rps11 | GTR+I | 37569-37982, 51151-51603 |
| rpl20, rpoC2 | GTR+I | 37983-38353, 46867-51150 |
| rpl22 | GTR+G | 38354-38748 |
| rpl23 | GTR+I | 38749-39042 |
| rpl2 | GTR+I | 39043-39872 |
| rpl32, rps15 | GTR+G | 39873-40053, 52299-52583 |
| rpl33, rpoA, rps19, rps8 | GTR+I | 40054-40266, 40381-41450, 52917-53201, 55812-56216 |
| rps12, rps7 | GTR+I | 51604-51984, 55224-55811 |
| rps3 | GTR+I | 53913-54611 |
| ycf1 | GTR+I | 56217-62965 |
| ycf2 | GTR+I | 62966-70599 |
| ycf3 | GTR+I | 70600-71112 |
