# Supplementary material for: Phylogeny, Age, and Evolution of Tribe Lilieae (Liliaceae) Based on Whole Plastid Genomes
Source: Front Plant Sci. 2022 Feb 1;12:699226. doi: 10.3389/fpls.2021.699226 (PMC8845482; doi:10.3389/fpls.2021.699226)
Supplement: Supplementary file 8 [file Table_4.DOCX]

**Supplementary table 4 |** Priors and mean ages used to calibrate the Liliales phylogenomic tree (see Fig. 2).

| Node | Mean | s.d. or Range | Application |
| --- | --- | --- | --- |
| Alstroemeriaceae *Luzuriaga* crown | 23.2 | 0.5 | Iles et al., 2015; Huang et al., 2018; Mennes et al. 2015 |
| Ripogonaceae stem | 51.5 | 0.5 | Iles et al., 2015; Huang et al., 2018; Mennes et al. 2015 |
| Zingiberales crown | 83 | 0.5 | Iles et al., 2015; Givnish et al., 2018; Xie et al., 2020 |
| Smilacacea *Smilax china* clade stem | 6.265 | 7.2-5.33 | Chen et al., 2014 |
| Smilacacea *Smilax* crown | 46 | 54.8-37.2 | Huang et al., 2018; Mennes et al. 2015 |
| Liliales stem | 123.8 | 131.1-115.6 | Givnish et al., 2018 |
