# Supplementary material for: Phylogeny, Age, and Evolution of Tribe Lilieae (Liliaceae) Based on Whole Plastid Genomes
Source: Front Plant Sci. 2022 Feb 1;12:699226. doi: 10.3389/fpls.2021.699226 (PMC8845482; doi:10.3389/fpls.2021.699226)
Supplement: Supplementary file 9 [file Table_5.DOCX]

**Supplementary table 5 |** Seven characters (number lines) and bulb states (letter line) used in this study.

| Species | Bulbs | | stem height (cm) | leaf length (cm) | leaf width(cm) | flower number | tepal length (cm) | tepal width (cm) |
| --- | --- | --- | --- | --- | --- | --- | --- | --- |
|  | component number | bulb type^*^ |  |  |  |  |  |  |
| *C. cathayanum* | 5-35 | C | 50-150 | 10-22 | 6-16 | 3-5 | 13-15 | 1.5-2 |
| *C. cordatum* | 5-35 | C | 70-200 | 15-20 | 12-15 | 10-18 | 12-18 | 1.5-2 |
| *C. giganteum* | 5-45 | C | 100-200 | 15-20 | 12-15 | 10-16 | 12-15 | 1.5-2 |
| *F. anhuiensis* | 1-10 | C | 10-50 | 10-15 | 0.5-3.5 | 1-4 | 3-5 | 1-1.5 |
| *F. cirrhosa* | 1-2 | A | 15-60 | 4-12 | 0.3-1.5 | 1-3 | 3-5 | 1.2-1.8 |
| *F. crassicaulis* | 1-2 | A | 30-60 | 7-10 | 1-2.6 | 1 | 4-5 | 1.1-1.2 |
| *F. dajinensis* | 1-2 | A | 20-50 | 3.5-11 | 0.2-1 | 1-4 | 1.8-2.3 | 0.5-0.6 |
| *F. davidii* | 40-120 | C | 10-33 | 3-5.5 | 2-2.8 | 1 | 3-4 | 0.7-1.4 |
| *F. delavayi* | 2-3 | A | 17-35 | 2-7 | 1-3 | 1 | 3.2-4.5 | 1.2-1.5 |
| *F. eduardii* | 2-3 | A | 30-65 | 15 | 2 | 4-6 | 6 | 2-3 |
| *F. fusca* | 1-2 | A | 8-22 | 9-3.3 | 0.7-2 | 1 | 1.6-1.8 | 0.6-0.7 |
| *F. karelinii* | 1-2 | A | 12-35 | 5-10 | 1 | 1-8 | 1-1.5 | 0.3-0.5 |
| *F. maximowiczii* | 20-60 | C | 27-54 | 4.5-10 | 0.3-1.3 | 1-2 | 3.5-4 | 0.4-1.4 |
| *F. meleagroides* | 2-3 | A | 20-40 | 5-15 | 0.1-0.5 | 1 | 2-3.5 | 0.5-0.8 |
| *F. monantha* | 1-2 | A | 45-60 | 10-12 | 1.5-4.5 | 1 | 4.5-5 | 1.5 |
| *F. pallidiflora* | 1-2 | A | 30-60 | 5-12 | 1-3 | 1-4 | 3.4-4.5 | 1.2-1.6 |
| *F. persica* | 2-3 | A | 50-60 | 8-12 | 0.9-1.8 | 10-22 | 1.2 | 0.6 |
| *F. przewalskii* | 1-2 | A | 20-40 | 3-7 | 0.3-04 | 1-2 | 2-3 | 0.6-0.7 |
| *F. sichuanica* | 1-2 | A | 20-50 | 3-14 | 0.2-0.8 | 1-3 | 2.5-4 | 0.5-1.3 |
| *F. sinica* | 1-2 | A | 30 | 3-8 | 0.5-2 | 1-2 | 2.5-4.5 | 1.3-2 |
| *F. taipaiensis* | 1-2 | A | 30-40 | 5-10 | 0.3-1.2 | 1 | 3-4 | 0.9-1.2 |
| *F. thunbergii* | 1-2 | A | 50-80 | 7-11 | 1-2.5 | 1-6 | 2.5-3.5 | 1 |
| *F. tortifolia* | 1-2 | A | 20-100 | 5-5.5 | 0.8-2 | 1 | 3 | 1-2 |
| *F. unibracteata* | 1-2 | A | 15-40 | 3.6-5.5 | 0.3-0.5 | 1 | 2.5-2.7 | 0.6 |
| *F. ussuriensis* | 1-2 | C | 100 | 7-14 | 0.3-0.65 | 1-3 | 3.5 | 1.5 |
| *F. verticillata* | 1-2 | A | 40-50 | 5-9 | 0.2-0.6 | 1-5 | 2-3 | 1-1.5 |
| *F. walujewii* | 1-2 | A | 20-40 | 5.5-10 | 0.2-0.9 | 1 | 3.5-4.5 | 1.2-1.4 |
| *F. yuminensis* | 1-2 | A | 30-50 | 5-5.5 | 0.8-2 | 1-12 | 1.5-2.2 | 0.6-1.5 |
| *F. yuzhongensis* | 1-2 | A | 20-50 | 3-8 | 0.2-0.6 | 1-2 | 2-4 | 0.6-1.8 |
| *L. amabile* | 15-25 | B | 40-80 | 2-7.5 | 0.5-0.8 | 1-3 | 3.5-5 | 0.8-1 |
| *L. anhuiense* | 60-100 | B | 60-120 | 8-14 | 0.7-1.1 | 2 | 16 | 3-4 |
| *L. bakerianum* | 24-60 | B | 60-90 | 4-7.5 | 0.4-0.7 | 1-3 | 6.5-8.3 | 1.4-1.8 |
| *L. brownii* | 40-120 | B | 70-200 | 7-15 | 0.6-2 | 1-4 | 13-18 | 2-4.3 |
| *L. bulbiferum* | 20-40 | BC | 20-120 | 10 | 1 | 1-5 | 4-6 | 0.3-0.7 |
| *L. callosum* | 20-30 | B | 50-90 | 6-10 | 0.3-0.5 | 1-5 | 3-41 | 0.4-0.6 |
| *L. candidum* | 80-160 | B | 120 | 3-18 | 0.8-2.2 | 2-20 | 6.2 | 1.2 |
| *L. cernuum* | 15-25 | B | 65 | 8-12 | 0.2-0.4 | 1-6 | 3.5-4.5 | 0.8-1 |
| *L. pensylvanicum* | 25-43 | B | 50-70 | 4-5 | 3-4 | 1-2 | 7-9 | 1.5-2.3 |
| *L. davidii* | 20-36 | B | 50-100 | 7-12 | 0.2-0.6 | 1-8 | 5-6 | 1.2-1.4 |
| *L. distichum* | 70-110 | B | 60-120 | 8-15 | 2-4 | 2-12 | 3.5-4.5 | 0.6-1.3 |
| *L. duchartrei* | 20-26 | B | 50-85 | 4.5-5 | 1 | 1-9 | 4.5-6 | 1.2-1.4 |
| *L. fargesii* | 12-20 | B | 20-70 | 10-14 | 2.5-5 | 1-6 | 3-3.5 | 0.7-1 |
| *L. formosanum* | 30-60 | B | 20-55 | 10-12 | 0.4-0.7 | 1-10 | 11.5-14.5 | 2.2 |
| *L. hansonii* | 20-40 | B | 100-150 | 10-18 | 2-4 | 4-12 | 3-4 | 1-1.5 |
| *L. henricii* | 20-40 | B | 60-120 | 12-15 | 9-14 | 5-6 | 3.5-5 | 1.2-2 |
| *L. henryi* | 65-80 | B | 100-200 | 2-15 | 1.5-2.7 | 2-12 | 5-7 | 2 |
| *L. japonicum* | 25-35 | B | 30-90 | 10.8-27 | 0.4-1.8 | 1-3 | 10-15 | 2-4 |
| *L. lancifolium* | 25-35 | BC | 80-150 | 6.5-9 | 1-1.8 | 3-11 | 6-10 | 1-2 |
| *L. lankongense* | 18-26 | B | 40-150 | 3-10 | 0.5-1.7 | 1-4 | 5-5.5 | 0.8-1 |
| *L. leichtlinii* | 20-30 | B | 50-200 | 3-10 | 0.6-1.2 | 2-8 | 4.5-6.5 | 0.9-1.5 |
| *L. leucanthum* | 110-76 | B | 60-150 | 8-17 | 0.6-1 | 2-4 | 12-15 | 1.6-2.8 |
| *L. longiflorum* | 30-50 | B | 45-90 | 20-25 | 0.8-1.2 | 1-2 | 13-18 | 2.5-4 |
| *L. lophophorum* | 14-18 | B | 10-45 | 5-12 | 0.3-2 | 1-3 | 4.5-5.7 | 0.9-1.6 |
| *L. martagon* | 26-46 | B | 45-90 | 6.5-11 | 1-2 | 2-7 | 3.2-3.8 | 0.8-0.9 |
| *L. matangense* | 10-20 | B | 23-35 | 6-11 | 0.1-0.4 | 1 | 2.5-3.5 | 0.5-0.7 |
| *L. nanum* | 10-20 | B | 10-30 | 4-8.5 | 0.2-0.4 | 1 | 2.5-2.7 | 1-1.2 |
| *L. nepalense* | 46-66 | B | 40-120 | 5-16 | 0.8-5.5 | 1-5 | 6-9 | 1.6-1.8 |
| *L. pardalinum* | 40-80 | B | 280 | 4.9-26.5 | 0.3-5.6 | 1-35 | 3.5–10.4 | 0.9–2.2 |
| *L. pardanthinum* | 18-34 | B | 25-90 | 2.5-4.5 | 0.7-1.5 | 1-3 | 2.5-3 | 1.2-1.5 |
| *L. philadelphicum* | 50-70 | B | 120 | 2.9-10.2 | 0.3-2.3 | 1-6 | 4.5–7.7 | 2–3.2 |
| *L. primulinum* | 60-80 | B | 60-200 | 3-12 | 0.8-1.4 | 4-9 | 3-9 | 1-1.7 |
| *L. pumilum* | 20-32 | B | 15-60 | 3.5-9 | 1.5-3 | 1-12 | 4-4.5 | 0.8-1.1 |
| *L. regale* | 50-70 | B | 50 | 6-8 | 0.2-0.3 | 1-19 | 9-11 | 1.5-2 |
| *L. rosthornii* | 50-70 | B | 40-100 | 8-15 | 0.8-1 | 1-9 | 6-6.5 | 0.9-1.1 |
| *L. sargentiae* | 40-70 | BC | 45-160 | 5.5-12 | 1-3 | 1-4 | 14-17 | 2-2.8 |
| *L. speciosum* | 25-45 | B | 60-120 | 2.5-10 | 2.5-4 | 1-5 | 6-7.5 | 1-2 |
| *L. sulphureum* | 40-70 | BC | 80-120 | 7-13 | 1.3-3.2 | 2 | 17-19 | 1.8-2.2 |
| *L. superbum* | 50-70 | B | 120-280 | 7.1-26.1 | 0.7-2.7 | 1-22 | 6.8-10.5 | 1.1-2.1 |
| *L. taliense* | 20-30 | B | 70-150 | 8-10 | 0.6-0.8 | 2-13 | 4.5-5 | 1 |
| *L. tsingtauense* | 20-60 | B | 40-85 | 7-15 | 1.6-4 | 1-7 | 4.8-5.2 | 1.2-1.4 |
| *L. washingtonianum* | 30-50 | B | 200 | 3.7-12.3 | 0.9-4.7 | 1-33 | 6.7-11.3 | 0.9-1.7 |
| *L. stewartianum* | 13-21 | B | 35-55 | 4-8 | 0.2-0.3 | 1-2 | 3.5 | 0.6 |
| *L. saluenense* | 20-32 | B | 30-90 | 3.5-7 | 0.8-1.5 | 1-7 | 3.5-5.2 | 1.6-2 |
| *L. farreri* | 20-30 | B | 25-75 | 3-9 | 0.5-0.8 | 1-2 | 2.5- 3.5 | 2.2- 3 |
| *L. gongshanense* | 18-30 | B | 50 | 4 | 0.6 | 1-2 | 4.0-4.4 | 2.0-2.2 |
| *L. meleagrinum* | 20-36 | B | 35-100 | 4.5-11 | 0.8-3.5 | 2-4 | 4-5 | 1.8-2.5 |
| *N. bulbuliferum1* | 30-60 | C | 60-150 | 10-18 | 1-2 | 10-24 | 2.5-3.8 | 0.8-1.2 |
| *N. bulbuliferum2* | 30-60 | C | 60-100 | 10-20 | 1-2.5 | 10-16 | 3.5-5 | 1-2 |
| *N. macrophyllum* | 10-40 | C | 20-35 | 6-15 | 0.4-0.8 | 2-6 | 2.5-5 | 0.6-1.5 |

*: A: few scales, B: many scales, C: numerous bulblets
