# Supplementary material for: Phylogeny, Age, and Evolution of Tribe Lilieae (Liliaceae) Based on Whole Plastid Genomes
Source: Front Plant Sci. 2022 Feb 1;12:699226. doi: 10.3389/fpls.2021.699226 (PMC8845482; doi:10.3389/fpls.2021.699226)
Supplement: Supplementary file 10 [file Table_6.DOCX]

**Supplementary table 6 |** Major characteristics of 80 Lilieae plastomes.

| Taxon | Total length (bp) | Total GC (%) | LSC length (bp) | IR length (bp) | SSC length (bp) |
| --- | --- | --- | --- | --- | --- |
| *C. cathayanum* | 152,416 | 37.10 | 82,355 | 26,378 | 17,305 |
| *C. cordatum* | 152,410 | 37.10 | 82,182 | 26,445 | 17,338 |
| *C. giganteum* | 152,653 | 37.10 | 82,345 | 26,500 | 17,308 |
| *F. anhuiensis* | 152,209 | 37.00 | 81,900 | 26,372 | 17,565 |
| *F. cirrhosa* | 151,998 | 37.00 | 81,756 | 26,349 | 17,544 |
| *F. crassicaulis* | 151,860 | 37.00 | 81,597 | 26,368 | 17,527 |
| *F. dajinensis* | 151,785 | 37.00 | 81,545 | 26,350 | 17,540 |
| *F. davidii* | 152,900 | 36.90 | 82,364 | 26,746 | 17,044 |
| *F. delavayi* | 151,914 | 37.00 | 81,652 | 26,356 | 17,550 |
| *F. eduardii* | 152,224 | 37.00 | 81,992 | 26,353 | 17,526 |
| *F. karelinii* | 152,118 | 36.90 | 81,872 | 26,390 | 17,466 |
| *F. maximowiczii* | 151,705 | 37.10 | 80,981 | 26,581 | 17,562 |
| *F. meleagroides* | 151,846 | 37.00 | 82,131 | 26,380 | 16,955 |
| *F. monantha* | 152,153 | 37.00 | 81,893 | 26,349 | 17,562 |
| *F. pallidiflora* | 152,078 | 37.00 | 81,779 | 26,391 | 17,517 |
| *F. persica* | 151,803 | 37.00 | 81,635 | 26,330 | 17,508 |
| *F. przewalskii* | 151,686 | 37.00 | 81,447 | 26,352 | 17,535 |
| *F. sichuanica* | 151,969 | 36.90 | 81,730 | 26,350 | 17,539 |
| *F. sinica* | 152,064 | 36.90 | 81,923 | 26,350 | 17,441 |
| *F. taipaiensis* | 151,707 | 37.00 | 81,452 | 26,352 | 17,551 |
| *F. thunbergii* | 152,155 | 37.00 | 81,891 | 26,350 | 17,564 |
| *F. tortifolia* | 152,005 | 37.00 | 81,777 | 26,361 | 17,506 |
| *F. unibracteata* | 151,971 | 36.90 | 81,730 | 26,350 | 17,541 |
| *F. ussuriensis* | 151,524 | 37.00 | 81,733 | 26,339 | 17,113 |
| *F. verticillata* | 151,959 | 37.00 | 81,729 | 26,362 | 17,506 |
| *F. walujewii* | 151,920 | 36.90 | 81,744 | 26,328 | 17,520 |
| *F. yuminensis* | 151,813 | 37.00 | 81,533 | 26,378 | 17,524 |
| *F. yuzhongensis* | 151,645 | 37.00 | 81,513 | 26,351 | 17,430 |
| *F. fusca* | 151,739 | 37.00 | 81,473 | 26,365 | 17,536 |
| *L. amabile* | 152,567 | 37.00 | 82,002 | 26,492 | 17,581 |
| *L. anhuiense* | 152,652 | 37.00 | 82,090 | 26,520 | 17,522 |
| *L. bakerianum* | 151,655 | 37.10 | 81,222 | 26,426 | 17,581 |
| *L. brownii* | 152,648 | 37.00 | 82,070 | 26,526 | 17,526 |
| *L. bulbiferum* | 152,686 | 37.00 | 82,084 | 26,492 | 17,618 |
| *L. callosum* | 152,626 | 37.00 | 82,041 | 26,491 | 17,603 |
| *L. candidum* | 152,101 | 37.00 | 81,482 | 26,488 | 17,643 |
| *L. cernuum* | 152,604 | 37.00 | 82,059 | 26,481 | 17,583 |
| *L. davidii* | 152,126 | 37.00 | 81,465 | 26,507 | 17,647 |
| *L. distichum* | 152,644 | 37.00 | 82,028 | 26,498 | 17,620 |
| *L. duchartrei* | 152,566 | 37.00 | 81,793 | 26,577 | 17,619 |
| *L. fargesii* | 153,235 | 36.90 | 82,218 | 26,990 | 17,037 |
| *L. farreri* | 152,024 | 37.00 | 81,859 | 26,439 | 17,287 |
| *L. formosanum* | 152,653 | 37.00 | 82,102 | 26,514 | 17,523 |
| *L. gongshanense* | 152,022 | 37.00 | 81,678 | 26,407 | 17,530 |
| *L. hansonii* | 152,655 | 37.00 | 82,052 | 26,492 | 17,619 |
| *L. henricii* | 152,784 | 37.00 | 82,430 | 26,411 | 17,532 |
| *L. henryi* | 153,119 | 37.00 | 82,481 | 26,553 | 17,532 |
| *L. japonicum* | 152,613 | 37.10 | 82,039 | 26,544 | 17,486 |
| *L. lancifolium* | 152,574 | 37.00 | 82,008 | 26,492 | 17,582 |
| *L. lankongense* | 152,611 | 37.00 | 81,996 | 26,555 | 17,505 |
| *L. leichtlinii* | 152,604 | 37.00 | 82,038 | 26,492 | 17,582 |
| *L. leucanthum* | 152,935 | 37.00 | 82,477 | 26,548 | 17,362 |
| *L. longiflorum* | 152,793 | 37.00 | 82,231 | 26,534 | 17,494 |
| *L. lophophorum* | 152,308 | 37.00 | 82,071 | 26,436 | 17,365 |
| *L. martagon* | 152,816 | 37.00 | 82,266 | 26,505 | 17,540 |
| *L. matangense* | 152,402 | 37.00 | 82,018 | 26,427 | 17,530 |
| *L. meleagrinum* | 152,278 | 37.00 | 81,849 | 26,411 | 17,607 |
| *L. nanum* | 152,349 | 37.00 | 81,989 | 26,431 | 17,498 |
| *L. nepalense* | 152,242 | 37.00 | 81,866 | 26,407 | 17,562 |
| *L. pardalinum* | 151,969 | 37.00 | 81,402 | 26,611 | 17,345 |
| *L. pardanthinum* | 152,718 | 37.00 | 82,151 | 26,520 | 17,527 |
| *L. pensylvanicum* | 152,052 | 37.10 | 81,484 | 26,492 | 17,584 |
| *L. philadelphicum* | 152,175 | 37.10 | 81,581 | 26,537 | 17,520 |
| *L. primulinum* | 152,306 | 37.00 | 81,955 | 26,394 | 17,563 |
| *L. pumilum* | 152,573 | 37.00 | 82,010 | 26,492 | 17,579 |
| *L. regale* | 152,996 | 37.00 | 82,371 | 26,548 | 17,529 |
| *L. rosthornii* | 152,613 | 37.00 | 82,279 | 26,271 | 17,792 |
| *L. saluenense* | 152,148 | 37.00 | 81,805 | 26,407 | 17,529 |
| *L. sargentiae* | 153,122 | 37.00 | 82,493 | 26,553 | 17,523 |
| *L. speciosum* | 152,597 | 37.10 | 82,036 | 26,544 | 17,473 |
| *L. sulphureum* | 153,099 | 37.00 | 82,473 | 26,553 | 17,520 |
| *L. superbum* | 152,069 | 37.00 | 81,403 | 26,619 | 17,428 |
| *L. taliense* | 153,055 | 36.90 | 82,459 | 26,543 | 17,510 |
| *L. tsingtauense* | 151,983 | 37.00 | 81,425 | 26,492 | 17,574 |
| *L. washingtonianum* | 151,967 | 37.10 | 81,394 | 26,611 | 17,351 |
| *L. xanthellum* | 152,307 | 37.00 | 81,922 | 26,427 | 17,531 |
| *N. bulbuliferum1* | 153,162 | 37.00 | 82,566 | 26,533 | 17,530 |
| *N. bulbuliferum2* | 153,150 | 37.00 | 82,570 | 26,526 | 17,528 |
| *N. macrophyllum* | 152,143 | 37.10 | 82,215 | 26,004 | 17,920 |
| *N. thomsonianum* | 152,588 | 37.10 | 82,222 | 26,434 | 17,498 |
