# Supplementary material for: Phylogeny, Age, and Evolution of Tribe Lilieae (Liliaceae) Based on Whole Plastid Genomes
Source: Front Plant Sci. 2022 Feb 1;12:699226. doi: 10.3389/fpls.2021.699226 (PMC8845482; doi:10.3389/fpls.2021.699226)
Supplement: Supplementary file 11 [file Table_7.DOCX]

**Supplementary table 7 |** Pagel’s λ and Blomberg’s K of seven morphological traits.

| State | λ | K |
| --- | --- | --- |
| bulb component number | 1.04 | 1.08 |
| stem height | 0.85 | 0.60 |
| leaf length | 0.51 | 0.31 |
| leaf width | 0.78 | 0.58 |
| flower number | 0.72 | 0.53 |
| tepal length | 0.82 | 0.56 |
| tepal width | 0.67 | 0.37 |
